# Molecular Transducers of Human Skeletal Muscle Remodeling under Different Loading States

**DOI:** 10.1016/j.celrep.2020.107980

**Published:** 2020-08-04

**Authors:** Tanner Stokes, James A. Timmons, Hannah Crossland, Thomas R. Tripp, Kevin Murphy, Chris McGlory, Cameron J. Mitchell, Sara Y. Oikawa, Robert W. Morton, Bethan E. Phillips, Steven K. Baker, Phillip J. Atherton, Claes Wahlestedt, Stuart M. Phillips

**Affiliations:** 1 Department of Kinesiology, McMaster University, Hamilton, ON, Canada; 2 Center for Therapeutic Innovation, University of Miami Miller School of Medicine, Miami, FL, USA; 3 School of Medicine, Royal Derby Hospital, University of Nottingham, Derby, UK; 4 Faculty of Kinesiology, University of Calgary, Calgary, AB, Canada; 5 School of Kinesiology and Health Studies, Queens University, Kingston, ON, Canada; 6 School of Kinesiology, University of British Columbia, BC, Canada; 7 Physical Medicine and Rehabilitation, Department of Medicine, McMaster University, Hamilton, Canada

**Keywords:** protein synthesis, atrophy, growth, untranslated region, skeletal muscle, hypertrophy, protein turnover, transcriptome, human, resistance exercise, unloading

## Abstract

Loading of skeletal muscle changes the tissue phenotype reflecting altered metabolic and functional demands. In humans, heterogeneous adaptation to loading complicates the identification of the underpinning molecular regulators. A within-person differential loading and analysis strategy reduces heterogeneity for changes in muscle mass by ∼40% and uses a genome-wide transcriptome method that models each mRNA from coding exons and 3′ and 5′ untranslated regions (UTRs). Our strategy detects ∼3–4 times more regulated genes than similarly sized studies, including substantial UTR-selective regulation undetected by other methods. We discover a core of 141 genes correlated to muscle growth, which we validate from newly analyzed independent samples (n = 100). Further validating these identified genes via RNAi in primary muscle cells, we demonstrate that members of the core genes were regulators of protein synthesis. Using proteome-constrained networks and pathway analysis reveals notable relationships with the molecular characteristics of human muscle aging and insulin sensitivity, as well as potential drug therapies.

## Highlights

• Muscle loading and unloading regulate the untranslated regions of mRNA
• Regulated genes form functional networks central to muscle plasticity
• 141 genes correlate with muscle growth in 3 independent validation cohorts
• Several growth-correlated network genes directly regulate myocyte protein synthesis

## Graphical Abstract

**Figure:**
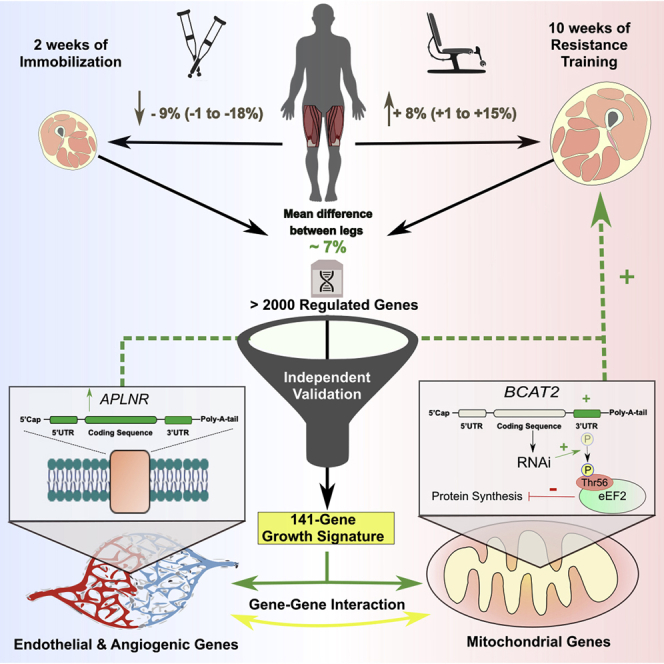

## Teaser

Stokes et al. identify and validate a core set of genes that are regulated in proportion to the magnitude of muscle protein turnover with loading. Several of these genes correlate with muscle growth only at their 3′ or 5′ untranslated region, and a subset directly influences protein synthesis *in vitro*.

## Introduction

Increased loading of skeletal muscle induces muscle fiber hypertrophy requiring the remodeling of myofibrillar and extracellular protein lattices. In contrast, unloading (UL) results in fiber atrophy, reductions in protein content, and a more fatigable tissue phenotype. In humans, voluntary loading (via resistance exercise training [RT]) leads to a highly heterogeneous physiological adaptation across individuals, which is associated with differential molecular response (Davidsen et al., 2011; Keller et al., 2011; Timmons et al., 2005). The implications of this heterogeneity are substantial, influencing muscle insulin sensitivity and age-related musculoskeletal frailty (Timmons et al., 2018; von Haehling et al., 2012) and potentially underpinning the compromised growth response in older individuals (Da Boit et al., 2016; Kosek et al., 2006). Less appreciated is the fact that the UL of human muscle also results in highly variable losses of muscle mass (Glover et al., 2008; Yasuda et al., 2005). The key regulators of heterogeneous muscle remodeling in response to loading and UL are unknown.

Collapsing data across individuals showing divergent physiological adaptation, as well as the use of small sample sizes, limits the reliable discovery of molecular regulators (Sweeney et al., 2017; Timmons, 2011). In contrast, accounting for physiological heterogeneity helps identify genes that regulate exercise adaptation in humans (Adami et al., 2018; Peter et al., 2014). Small-scale human physiology studies that focus on in-depth phenotyping limit reliable correlation modeling (Cohain et al., 2017; Schönbrodt and Perugini, 2013), particularly for genome-wide omics. The increasing use of omics has advanced our understanding of the molecular regulators of loading; however, proteomics applied to muscle tissue captures a very limited sample of the protein-coding genome (Camera et al., 2017), which limits the validity of pathway analysis (Timmons et al., 2015). Unbiased transcriptome profiling can be more reliable, and variation in RNA can explain up to 75% of the variation in protein abundance, with some exceptions (Li and Biggin, 2015). Nevertheless, it has been reported that RNA does not reliably predict changes in protein abundance during muscle loading (Robinson et al., 2017). Typically, RNA quantification relies on technology that averages across an otherwise complex set of transcripts from a gene (Shalek et al., 2013) or uses short-read RNA sequencing that misses significant proportions of the tissue transcriptome (Sood et al., 2016). Thus, suboptimal RNA profiling may contribute to a discordance between muscle RNA and protein abundance (Robinson et al., 2017).

**Figure 1:**
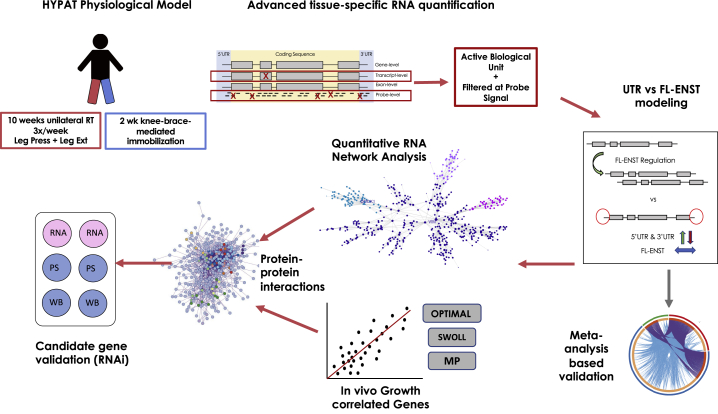
Experimental Workflow and Analysis Strategy We used a paired unilateral loading (10 weeks of progressive RT) and unloading (UL; 2 weeks of UL) model in combination with genome-wide transcriptomic analysis (Timmons et al., 2018) to study differential expression of gene UTRs and protein coding regions. Probes were subjected to extensive filtering before downstream analysis (see STAR Methods). Significance analysis of microarrays implemented in the R programming environment (SAMR) was used to detect significantly regulated genes (Tusher et al., 2001), which were then used as an input list for quantitative network analysis using the MEGENA package for R (Song and Zhang, 2015). We also determined which genes played a role in regulating dynamic lean tissue growth in independent cohorts (total n = 100). Highly co-regulated genes and growth-correlated gene lists were used as input in metascape.org and https://www.networkanalyst.ca to characterize protein-protein interaction networks and drug signatures.

We hypothesized that a method that reduced physiological heterogeneity to loading states and improved the fidelity of RNA quantification (Figure 1) would allow the improved identification of the molecular regulators of adaptation. We contrasted muscle subjected to a loading hypertrophic stimulus with the paired (contralateral) muscle subjected to UL to induce atrophy (a model called HypAt). The aim of the HypAt model is to measure molecular responses to loading and UL within an individual to reduce heterogeneity and more readily reveal potential regulators. We characterized phenotypic responses to HypAt and implemented an approach to quantify both full-length mRNA transcripts (FL-ENST) and the untranslated regions (UTRs) of the same “gene,” as they can regulate translational efficiency (Mayr, 2017). We established key genes regulated during gains in lean mass in three independent cohorts, some of which directly regulate protein synthesis *in vitro*. We used proteome-constrained network and pathway analyses to discover relationships between the transducers of dynamic muscle remodeling, muscle aging, and insulin resistance and match molecular signatures to potentially useful US Food and Drug Administration (FDA)-approved drugs.

## Results

### Muscle Growth and Integrated Myofibrillar Protein Synthesis

**Figure 2:**
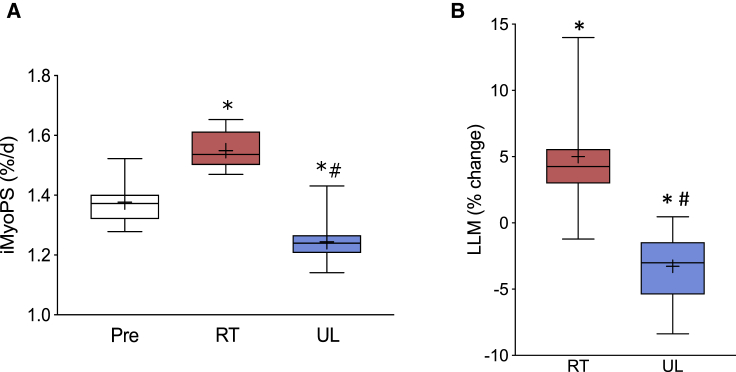
Dynamic Muscle Loading Alters Muscle Protein Synthesis (MPS) and Muscle Size (A) Absolute change in integrated myofibrillar protein synthesis rates (n = 12). (B) Percentage change in leg lean mass (LLM) after 10 weeks of unilateral RT and 2 weeks of UL, respectively (n = 12). ^∗^Statistically different from Pre (baseline value); #statistically different from RT value (p < 0.05). In both panels, the boxes include the 25^th^, 50^th^, and 75^th^ quartiles and whiskers represent the maximum and minimum values. The mean value is depicted by +. See also Figure S1.

Participant characteristics for the paired analysis (HypAt) are displayed in Table S1 (see STAR Methods for the demographics of the additional clinical studies). Dietary macronutrient intakes were stable during the study (17% ± 6% protein, 49% ± 15% carbohydrate, 32% ± 12% fat). The protein consumed was 1.5 ± 0.7 g ⋅ kg^−1^ ⋅ day and 1.7 ± 0.2 g ⋅ kg^−1^ on training days, sufficient to fully support muscle hypertrophy (Morton et al., 2018). At baseline, the daily step count averaged 9,900 ± 5,100 steps per day and remained unchanged throughout the study. The average changes in function were as expected—isometric maximal voluntary contraction torque (ISO-MVC) increased by 14% after RT (median: 5.6%; range: −2.5% to 50.1%; p < 0.05) and decreased by ∼14% following UL (median: −12.7%; range: −1.3% to −28.3%; p < 0.05). Leg extension strength increased by 48% (median: 46%; range: 13%–100%) and leg press by 67% after RT (median: 72%; range: 13%–158%; p < 0.05). Orally administered deuterated water and muscle biopsies quantified integrated myofibrillar protein synthesis (iMyoPS) rates (Wilkinson et al., 2014) (deuterium enrichment in saliva over time is shown in Figure S1E). Pre-intervention iMyoPS was 1.37% ± 0.07% day^−1^, consistent with earlier work (Brook et al., 2016). iMyoPS increased 12.6% after 10 weeks of RT and decreased 9.5% with 2 weeks of UL (p < 0.05; Figure 2A). In HypAt, leg lean mass (LLM; by dual-energy X-ray absorptiometry [DXA]) increased by ∼5% (median: 4.2%; range: −1.2% to 14.0%; p < 0.05) in response to RT, while the immobilized leg demonstrated a 3.4% decrease (median: −3.0%; range: 0.5 to −8.4%; p < 0.05; Figure 2B). In addition, we found, using magnetic resonance imaging, that mid-thigh *vastus lateralis* cross-sectional area (VLCSA) increased by 8.1% following RT (median: 8.2%; range: 0.9%–15%; p < 0.05), whereas VLCSA was reduced in the UL leg by 9.0% (median: −6.8%; range: 0.4 to −18.5%; p < 0.05; Figure S1A). Quadriceps volume (Figure S1B) and peak quadriceps CSA (Figure S1C) demonstrated a similar pattern. Calculating the individual physiological responses, as proposed (HypAt), reduced the inter-subject heterogeneity for change in muscle size by almost half (coefficient of variation [CoV] was 90%, 88%, and 49% for RT, UL, and HypAt, respectively). Thus, each subject provided a more consistent differential change in muscle mass, and hence improved the validity of any means-based analyses.

### Functional RNA Networks Are in High Agreement with Established Proteome Responses to Exercise

**Figure 3:**
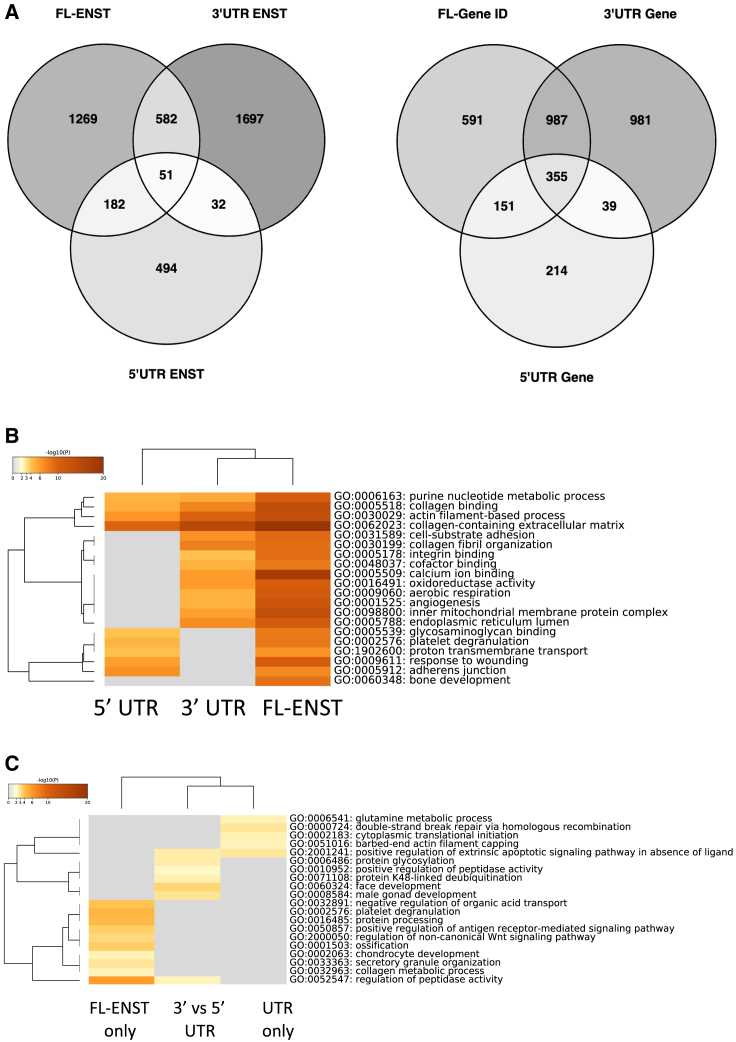
The Untranslated Regions (UTRs) of Genes Are Subject to Extensive Regulation by Dynamic Muscle Loading States (A) Venn diagrams show the extent of overlap in FL-ENST, 3′ UTR, and 5′ UTR gene expression. More genes showed regulation at the 3′ UTR than at the FL-ENST level; however, there was substantial overlap. (B and C) Heatmaps showing (B) functional pathway enrichment based on gene region (5′ UTR, 3′ UTR, or FL-ENST) and (C) the significance level of different Gene Ontology pathways by transcript type: FL-ENST only versus UTR only versus differential 3′ UTR/5′ UTR regulation only. The ontology enrichment scores are relatively modest after correction for tissue and platform bias. Heatmaps were generated using Metascape (metascape.org). The colors indicate the level of significance, with the darker colors being more significant. The gray boxes are non-significant results.

Global muscle transcriptomes were generated from four independent clinical studies using the Affymetrix HTA 2.0 array. A probe set signal, based on ensemble identifiers (Dai et al., 2005), either represented a FL-ENST or the 5′ UTR or the 3′ UTR signal from a FL-ENST. For FL-ENST versus 3′ UTR comparisons, there were 32,307 probe sets, while for FL-ENST versus 5′ UTR, there were 28,291. This resulted in 44,358 largely protein-coding probe sets, representing 11,628 genes. For FL-ENST differential analysis, 18% of the 11,628 genes had at least 1 FL-ENST regulated (false discovery rate [FDR] <5%, fold change [FC] >1.2; 1,435 upregulated and 649 downregulated; Data S1). There were more genes regulated by measuring their 3′ UTR than the FL-ENST (Figure 3A; Data S1). There were 1,162 upregulated and 1,200 downregulated 3′ UTR (fewer 5′ UTRs were regulated; 553 genes upregulated and 206 downregulated; Data S1). Compared with clinical studies using interventions of similar duration (Damas et al., 2019; Melov et al., 2007; Phillips et al., 2013; Raue et al., 2012), our approach identified ∼4 times more regulated molecular events. The regulated groups of genes had the expected roles in extracellular matrix remodeling, mitochondrial biology, and angiogenic processes (Figure 3B; Data S1).

The measurement of changes in the UTR signal allows for the identification of factors, beyond mRNA abundance, that influence translation. For example, a greater 3′ UTR:protein coding sequence (CDS) ratio can lead to lower protein production (Floor and Doudna, 2016; Kocabas et al., 2015), presumably reflecting increased miRNA and RNA-binding protein target sites within the lengthened 3′ UTR. For HypAt upregulated genes, the pattern was >70% consistent between FL-ENST and the UTR signals; however, this consistency was reduced to 40% for downregulated genes (Data S1). There were >1,000 genes that were statistically “regulated” only at their UTR signal (Figure 3A; Data S1). We applied a heuristic method (see Method Details) to identify genes that demonstrated a distinct pattern of regulation across the 3 signals, revealing a subset of FL-ENST (n = 112 genes), 3′ UTR (n = 200 genes), and 5′ UTR transcripts (n = 62 genes), which demonstrated selective differential regulation *in vivo* (e.g., FL-ENST upregulated with reduced 5′ UTR signals or 3′ UTR signals reduced more than the FL-ENST signal, indicating a shift in the mRNA population; Data S1). For example, seven genes involved in the repair of double-strand DNA breaks (*HUS1B*, *LIG3*, *MRNIP*, *RAD51C*, *RIF1*, SPIDR, and *ZFYVE26*) had unchanged levels of mRNA, yet their 3′ UTR signal was substantially reduced. The 3′ UTR of branched-chain amino acid transaminase 2 (*BCAT2*), a mitochondrial branched-chain amino acid catabolism gene, increased on average, indicating that the BCAT2 population of mRNAs in muscle had longer 3′ UTRs during gains in lean mass. Furthermore, there were 275 discordantly regulated genes at the 3′ UTR versus 5′ UTR (Data S1). The biological processes carried out by these genes included developmental and peptide or K48-linked de-ubiquitination (Figure 3C).

We used quantitative network modeling (Song and Zhang, 2015) to demonstrate that the HypAt-regulated genes formed significant gene expression networks (Data S1). This method identifies groups of co-regulated genes and does so independently of any knowledge of gene function (i.e., it is data driven). The identification of known gene-gene relationships provides internal validation and a framework to assign genes of unknown function (van Dam et al., 2015). Analysis of the FL-ENST yielded networks centered around mitochondrial genes (e.g., NDUFS3, reflecting tight coordination of the mitochondrial transcriptome; Figure S2). The significant ontology categories, beyond tissue bias (Timmons et al., 2015), included Gene Ontology (GO):0055114 “oxidation-reduction process” (p < 1 × 10^−15^) for the *NDUFS3* network. Another major network centered around Dedicator of Cytokinesis 1 (*DOCK1*) (a 312-gene network; Figure S2), a Rho guanosine triphosphatase (GTPase)-related gene regulated by mechanical strain (Copley Salem et al., 2018) and genetically linked to cachexia (McDonald et al., 2017). The top ontology category of the *DOCK1* network was GO:0040011 “self-propelled movement of a cell” (p < 1 × 10^−11^). These analyses indicate that the molecules that we identified as regulated in the HypAt model reflect functional networks central to muscle tissue physiology, including many of the same features identified using proteome analysis (Robinson et al., 2017).

### Proteome-Constrained Network Modeling Reveals Growth-Regulating Pathways

Genes regulated in a manner correlated with net muscle growth may be responsible for inter-subject variation in exercise-induced muscle mass. We used 3 independent studies (n = 100 individuals, 200 HTA 2.0 gene chips; Data S1), each with DXA-measured changes in LLM following exercise training (Mitchell et al., 2014; Morton et al., 2016; Phillips et al., 2017) to establish which of the ∼2,000 HypAt-regulated genes were potential regulators of growth. For reliable estimates, sample sizes of ≥30 are required for correlation analysis (Gobbi and Jurman, 2015; Schönbrodt and Perugini, 2013). We calculated pre-post training differential probe set signal and Spearman rank correlation across each study and then aggregated consistent (directionality) correlation coefficients and found that 141 genes were correlated in a consistent manner with muscle mass gains (either at the FL-ENST, 3′ UTR or 5′ UTR level) and were regulated in the HypAt model. Genes correlating with muscle growth coded for proteins involved in purine ribonucleoside monophosphate biosynthesis (e.g., AMP, guanosine monophosphate [GMP], inosine monophosphate [IMP], and xanthosine monophosphate [XMP]), mitochondrial biology (Figure S3, inset) and included numerous positive regulators shown previously, such as the apelin receptor (*APLNR*, upregulated in HypAt, correlation coefficient (CC) = 0.34), a known mediator of hypertrophy (Hwangbo et al., 2017; Vinel et al., 2018). *APLNR* negatively co-varied with known negative regulators of hypertrophy (*FOXO3*, CC −0.4; *PRKAA2*, CC −0.3), which were negatively correlated with changes in LLM (CC −0.33 and −0.21, respectively). These in turn were strongly co-regulated,during muscle growth,with *KDR*, *TIE1*, and *NRP1*, which are potent regulators of angiogenesis.

Examination of the relationships between modulation of transcript 3′ UTR signals and the change in lean mass revealed several regulators of muscle growth (Data S1). The *BCAT2* 3′ UTR signal was strongly correlated (CC = 0.37, n = 100) to an increase in muscle mass, in which a greater *BCAT2* 3′ UTR signal means that more individual mRNAs had longer 3′ UTRs. This was in the absence of any relationship between the coding region signal and change in muscle mass. Another 3′ UTR-regulated gene, *TPI1* (triosephosphate isomerase 1), encodes a protein that plays a role in regulating the cytosolic redox state and is genetically linked to a muscle-wasting phenotype (Gnerer et al., 2006). Some 5′ UTR signals were also related to muscle growth (Data S1)—for example, the increase in 5′ UTR signal for exostosin glycosyltransferase 1 (*EXT1*) was strongly related to gain in lean mass (CC = 0.38, n = 100). *EXT1* is required for the biosynthesis of heparan sulfate, which in turn appears to be an essential factor for muscle remodeling and growth (Ghadiali et al., 2017).

One way to refine database-driven network analysis is to constrain the growth-correlated RNA networks by a muscle tissue-enriched proteome and model protein-protein interactions (PPIs) (https://www.networkanalyst.ca). Tissue-enriched PPI analysis of the growth-correlated RNAs (Figure S3; Data S1) yielded a list of pathways with known roles in cellular growth (Table 1), including ubiquitin protein ligase genes, apoptotic processes, and negative regulators of the nitrogen compound metabolic process. For example, *FOXO3* (negatively correlated with LLM changes during exercise training) was part of a PPI that contained 45 other *FOXO* signaling pathway members (Kyoto Encyclopedia of Genes and Genomes [KEGG] database, 1 × 10^−14^ FDR) and is a known regulator of cell growth (Sandri et al., 2013). The subset of genes found to be differentially regulated (Figure 3) and correlated with muscle growth are listed as an input file (Data S2) to allow for the generation of a 3-dimensional (3D) version of the proteome-constrained network model in Figure S3 (see Table S2 for instructions). The present analyses demonstrate that when measured in a sensitive and sophisticated manner, human muscle growth and dynamic changes in protein synthesis are accurately reflected in the pattern of responses observed in the human muscle transcriptome.

**Table 1:** Combining the Skeletal Muscle-Specific Proteome with the Core Transcriptional Signature from HypAt that Covaried with Gains in Muscle Mass across 3 Independent Studies Identified the Majority of Known Canonical Regulations of Cell Hypertrophy from Model Systems

Pathway	FDR
FOXO signaling	3E−14
MAPK signaling	3E−10
Neurotrophin signaling	1E−9
Mitophagy	6E−9
HIF-1 signaling	5E−8
Longevity regulating pathway	2E−7
AMPK signaling	3E−7
Hippo signaling	9E−7
PI3K-Akt signaling	1E−6
Apelin signaling	1E−6
Adipocytokine signaling	2E−6

FDR is −log value. FDR, false discovery rate.

### Identified Growth-Correlated Network Genes Directly Regulate Human Myocyte Protein Synthesis

**Figure 4:**
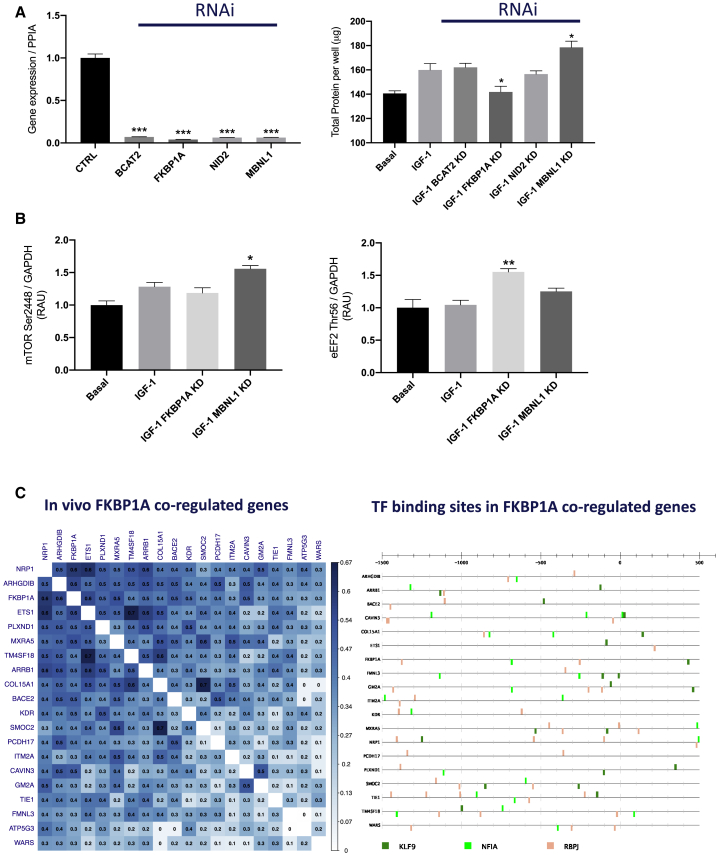
Growth-Regulated Genes Modulate Protein Synthesis and Anabolic Signaling in Human Muscle Cells RNAi targeting of individual members of the muscle mass-related gene network. (A) mRNA expression of BCAT2, FKBP1A, NID2, and MBNL1 relative to their own control (100%) following treatment with a pool of multiple siRNAs targeting each gene (BCAT2, FKBP1A, NID2, and MBNL1), with IGF-1 used to increase primary muscle cell protein synthesis. *p<0.05, **p<0.01, ***p<0.001. (B) Calculation of relative arbitrary units (RAUs) for mTOR Ser2448 and eEF2 Thr56, using phosphospecific antibodies (IGF-1 treatment in primary muscle cell in the presence or absence of FKBP1A and MBNL1 RNAi). *p<0.05, **p<0.01, ***p<0.001. (C) Correlation matrix of the *in vivo changes* in gene expression covarying with the change in FKBP1A. All of the genes were also correlated with exercise training-induced alterations in muscle lean mass (see Method Details). Transcription factor binding site enrichment analysis (−1,500 to +500 nt from the start codon using CiiiDER and controlled for bias in the muscle transcriptome) revealed that 3 transcription factors (KLF9, NFIA, and RBPJ) potentially coordinate this FKBP1A-angiogenesis related transcriptional “module” (i.e., they are not enriched in the larger lean-mass growth-related transcriptional signature).

We used puromycin-based measures of myocyte protein synthesis, in the presence of insulin growth factor 1 (IGF-1), with and without small interfering RNA (siRNA)-mediated downregulation of 4 genes identified from the list of regulated mRNA or 3′ UTR ENSTs that correlated with lean mass gains *in vivo* (*NID2*, *FKBP1A*, *BCAT2*, and *MBNL1*). As can be observed in Figure 4A, RNA interference (RNAi) effectively reduced RNA expression by >90% in differentiated myotubes after 3 days of treatment. There appeared to be some interaction between the loss of one gene and the expression of a second candidate regulator of muscle growth (Figure S4; see Discussion). In our analysis, the 3′ UTR signal of *FKBP1A* increased 20% in HypAt, and the change in 3′ UTR of the transcript variant ENST00000400137 strongly correlated with gains in lean mass *in vivo* (R = 0.31, n = 100). Knockdown (KD) of *FKBP1A in vitro* resulted in a reduction in puromycin signal, accompanied by an increase in eukaryotic elongation factor 2 (eEF2) phosphorylation (Figures 4B and S5) at Thr56 (the less active/inactive form), while the mammalian target of rapamycin (mTOR) Ser2448 (Figure 4B), P70 S6K1 Thr389, and 4E-BP1 Thr37/46 were not significantly altered (Figures S5 and S6). While the coding region of *FKBP1A* is clearly the “functional” entity, the importance of its regulation by altered 3′ UTR is further supported by the fact that it strongly covaried, with changes in >200 genes involved in extracellular matrix remodeling *in vivo* (bidirectionally), specifically with their 3′ UTR signals and not the signal from the entire mRNA (Data S1).

A core set of genes were both differentially regulated and quantitatively related to gains in lean mass, and there was evidence that these genes shared a number of common transcription factor binding sites (for KLF9, NF1A, and RBPJ) enriched (Gearing et al., 2019) over and above muscle-expressed genes or all HypAt-regulated genes (Figure 4C). The additional three genes chosen for RNAi targeting also validated our *in vivo* modeling. The loss of our proposed uncharacterized inhibitor of lean mass gains, MBNL1, was associated with an increase in protein synthesis and increased phosphorylation of mTOR Ser2448 (Figures 4A and 4B) and total protein (Figure S6). NID2—positively associated with lean mass gains—was effectively knocked down *in vitro* (Figures 4A and S7A), and this reduced global protein synthesis at 4 h (Figure S7B) and modestly suppressed total RNA (presumably rRNA), but not total protein content. Phosphorylation of p70 S6K1 and 4E-BP1, in response to IGF-1 treatment, was reduced when the *NID2* transcript was targeted (Figure S7D). The near-elimination of *BCAT2* expression also, as predicted, suppressed IGF-1-mediated protein synthesis at 4 h (Figures S7E and S7F) and reduced the phosphorylation of p70 S6K1 (Figure S7H), but it did not alter the total cell protein. It also resulted in an increase in eEF2 Thr56 phosphorylation (consistent with the inhibition of translation).

## Discussion

Implementing a within-person loading and analysis strategy facilitated the identification of the genome-wide molecular analysis of human muscle remodeling in a small human cohort. Combined with an updated RNA profiling strategy (to quantify 3′ or 5′ regulatory ends of protein coding mRNA) enabled us to identify molecular transducers of muscle growth. A subset of transcripts was found to be regulated in proportion to the magnitude of human muscle growth, including members of validated growth and atrophy canonical pathways. We established that four of these genes regulated muscle protein synthesis (MPS) and canonical signaling events that regulate translational control. Overall, our analyses support the utility of using human models to identify relevant molecular transducers of muscle activity while reinforcing the view of Sweeney et al. (2017), and our own experience, that integrating multiple independent clinical datasets and accounting for variations in phenotypic adaptability are crucial for producing reliable molecular models of human physiology.

### A Robust Molecular Model of Human Muscle during Changes in Mass

Many variables influence muscle responses to loading—age, biological sex, diet, and genetic variation (Roberts et al., 2018; Silventoinen et al., 2008). A within-subject study design enhanced the means-based statistical analysis and resulted in a far greater number of detected differentially expressed genes than comparably sized studies (Damas et al., 2019; Melov et al., 2007; Phillips et al., 2013; Raue et al., 2012). Our transcriptional analyses identified many genes with established roles in cell growth, but we discovered that they were regulated both by mRNA abundance and through differential modulation of their 5′ or 3′ UTR. Moreover, we demonstrate that UTR-specific regulatory events can covary with lean mass transitions in human muscle without corresponding changes to the full-length transcript. These regulatory events are missed by traditional transcriptomic analysis techniques that focus on the full transcript or gene-level regulation only. For instance, the recently curated database of transcriptional responses to acute and chronic exercise (Pillon et al., 2020) calculated only gene-level RNA regulation (from multiple different methods) and therefore erroneously considers many important growth-regulating genes as being unimportant in adaptation to exercise that we identified (e.g., *BCAT2*, *KDR*, *NRP1*). More generally, this type of database (Pillon et al., 2020) does not integrate physiological heterogeneity, and as such, any potential insights are obscured by the normalized means-based data analysis. In contrast, our unbiased analysis strategies yield a significant advance in our understanding of the molecular transducers of lean mass gain in response to RT.

Using our large multi-study tissue transcriptomic biobank and quantitative network analysis, we illustrated how HypAt-regulated genes may interact to influence skeletal muscle growth. Quantitative network analysis differs from database analysis as it reveals quantitative gene-gene interactions. In Figure S2, a multi-module mitochondrial network is presented that centers around well-recognized genes, such as *NDUFS3* and *SDHB*, but it also reveals the regulation of less well-studied genes—for example, *ChChd3*, a mitochondrial membrane protein (Darshi et al., 2011). Underscoring the power of this approach, quantitative network analysis correctly localized a recently discovered long non-coding RNA *LINC00116*, to our mitochondrial network (Figure S2). Two recent publications demonstrated that a peptide called mitoregulin, originating from *LINC00116*, localizes to the inner mitochondrial membrane and regulates mitochondrial complex I activity (Chugunova et al., 2019; Stein et al., 2018). In fact, many genes with established mitochondrial roles were positively correlated with the degree of muscle growth. For instance, the apelin receptor (*APLNR*) was regulated at the FL-ENST level. Apelin plays an important role in the sarcopenia of aging and positively regulates inter-myofibrillar mitochondrial content in mice (Vinel et al., 2018). We observed that *APLNR* expression was positively related to numerous mitochondrial genes (e.g., *ATP5G1*, *ATP5G3*, *BCAT2*, *COX7A2*, *NDUFB8*, *NDUFS2*) as part of the mitochondrial gene expression network (Figure S2). The HypAt approach efficiently identified molecular transducers of dynamic muscle remodeling, and when combined with validation in multiple independent studies, the analysis provides unrivaled detail of the genome-wide responses.

### Physiological Regulation of Human Muscle Protein Remodeling

The most conspicuous adaptation of protein turnover with immobilization-induced muscle UL is a rapid decline in MPS, first identified >30 years ago (Gibson et al., 1987) and that the loss of muscle mass can be explained completely by this reduction in rates of MPS (Phillips and McGlory, 2014). The analysis of the HypAt study demonstrated that reductions in iMyoPS represent a dominant feature of muscle mass changes during periods of disuse (Figure S1D), with muscle atrophy proceeding at a rate ∼5 times faster during UL than corresponding RT-induced muscle growth. Notably, while myofibrillar protein synthesis was enhanced with RT, the extent of upregulation was not correlated with muscle growth. Our molecular analysis demonstrated abundant regulation of mitochondrial and microvascular gene networks, which we speculate support adaptive changes and contribute to and support an increased MPS signal. It has been reported that mitochondria support angiogenesis, the coordinated migration and proliferation of endothelial cells to form new blood vessel branches (Diebold et al., 2019), and the question arises whether components of the mitochondria also influence muscle hypertrophy.

Mitochondrial transcript abundance is sensitive to muscle UL (Abadi et al., 2009; Powers et al., 2012; Timmons et al., 2006). Moreover, we have shown that maximal ADP-stimulated respiration is impaired within days of UL, but in the absence of detectable mitochondrial protein content (Miotto et al., 2019). A loss of mitochondrial function therefore appears to be an earlier manifestation of muscle disuse (Miotto et al., 2019), and this is directly reflected in the present transcriptome model. In addition to the mitochondrial-dominated gene network, numerous angiogenesis-related genes were differentially regulated in our study. The angiogenic program is stimulated early in an RT program and appears temporally coupled with muscle growth (Holloway et al., 2018). In the present study, not only was the regulation of *FKBP1A* linked to protein synthesis (it is a prolyl isomerase, but also binds the immunosuppressants rapamycin and FK506; Aylett et al., 2016) and its expression tightly coordinated with a number of key angiogenetic genes but it also covaried with the degree of muscle hypertrophy, possibly through the activities of KLF9, NFIA, and RBPJ (Figure 4C). Clearly, muscle growth and remodeling are multifaceted processes, and it is thus unsurprising that any relationship between muscle growth and the corresponding changes in iMyoPS would be complex, reflecting the precise composition of newly formed proteins.

### Leveraging Transcriptional Networks to Investigate Chronic Disease and Identify Potential Therapies

Immobilization impairs the sensitivity of the protein synthetic machinery to hyperaminoacidemia (Glover et al., 2008), implicating anabolic resistance as a key process in atrophy. Here, we find that the average *BCAT2* transcript has longer 3′ UTRs and that this change was positively related to gains in lean mass in three independent studies (without our distinct RNA method, the importance of *BCAT2* in this context would have been missed). BCAT is a mitochondrial protein that catalyzes the transamination of leucine to α-ketoisocaproic acid, leading to TOR complex 1 (TORC1) activation (Moghei et al., 2016). In our *in vitro* studies, loss of *BCAT2* in primary muscle cells resulted in reduced (Figure S7H) p70 S6K1 phosphorylation and protein synthesis (in response to IGF-1) and an increase in eEF2 Thr56 phosphorylation (also consistent with the inhibition of protein translation). In tumor cells, global shortening of 3′ UTRs is a hallmark of mTORC1 activation and increased protein production (Chang et al., 2015a). This implies, as previously posited (Phillips et al., 2013), that mTOR activation will not be key to explaining the variation in muscle growth across individuals. The loss of *BCAT2* expression *in vitro* also resulted in the cell reducing other members of the core 141-gene signature we describe that was regulated in proportion to changes in lean mass (i.e., *FKBP1A* and *NID2*; Figure S7A), as well as increased eEF2 Thr56 phosphorylation (consistent with direct FKBP1A KD; Figure 4B). Thus, the loss of BCAT2 signals to the cell to downregulate several other modulators of muscle growth or protein synthesis and suggests that leucine metabolism (Chawla et al., 1975; Escobar et al., 2010) and/or aspects of amino acid sensing in general regulate changes in human muscle mass in response to loading and UL.

**Figure 5:**
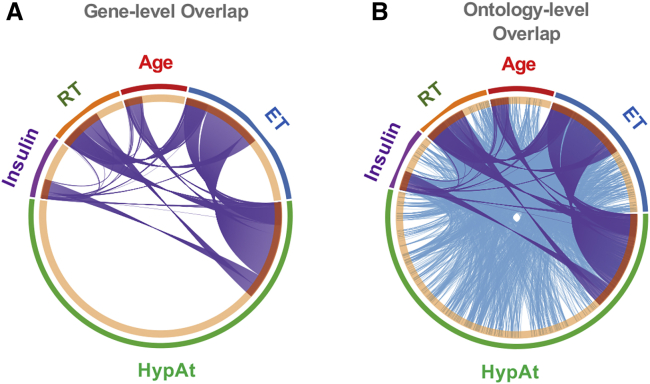
Genes Regulated by Potentially Related Physiological Conditions Show Substantial Pathway Overlap (A and B) Circos plots showing the overlap in gene expression (A) and Gene Ontology (B) between the HypAt model and additional biological signatures of potentially related physiological conditions (e.g., insulin sensitivity; RT [resistance training]; age; ET [endurance training]). While the overlap in individual genes is modest (HYPAT versus ET = 338 genes; HYPAT versus RT = 160 genes; HYPAT versus insulin sensitivity = 73 genes; HYPAT versus age = 69 genes; see Data S1 for full lists), the overlap at the pathway level is substantial (highlighting at least one caveat of relying on only gene identifiers to compare and contrast molecular profiles). Notably, there are pathway features of insulin sensitivity and aging that do not appear in the ET and RT signatures (obtained from healthy subjects).

**Figure 6:**
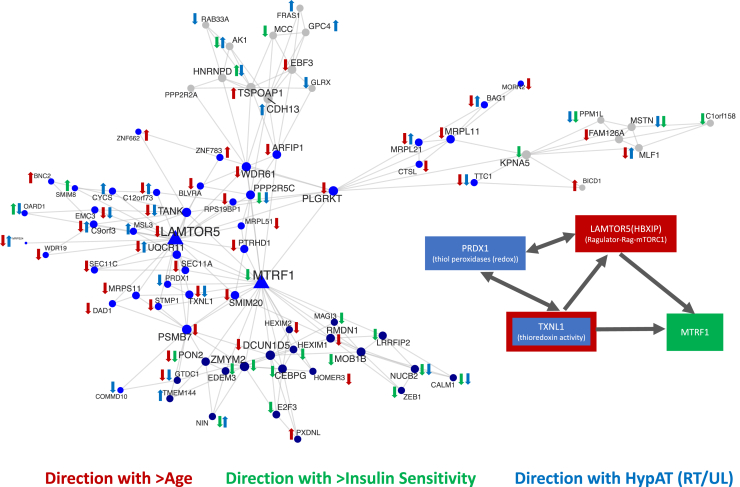
Quantitative Network Analysis Unveils Potentially Important Gene Interactions across Potentially Related Physiological Conditions An example network of gene interactions across different, yet interrelated physiological conditions, constructed using HypAt-, age-, and insulin sensitivity-regulated transcripts (FDR < 5%) as input into Megena (FDR < 1% Spearman correlation; p < 0.01 for module significance, p < 0.01 for network connectivity, and 10,000 permutations for calculating FDR and connectivity p values). This example network is centered on LAMTOR5, a gene encoding a subunit of the pentameric Ragulator complex involved in mTORC1 activation. From these interactome networks, relationships between genes can be discovered and subsequently studied in model systems. Triangle symbols represent ”hub genes,” whereas circles represent non-hub network members. Node colors represent different subnetwork clusters, and node size is proportional to node degree.

While our approach identified numerous known and validated genes, we also discovered several less well-characterized molecular transducers of muscle adaptation in humans. The main power of genome-wide transcript modeling is that the signatures generated can be used as powerful tools to investigate chronic disease and identify potential treatments (Chang et al., 2015b; Subramanian et al., 2017; Timmons et al., 2005). At the level of individual genes, shared features common to aging or insulin resistance and exercise have been, to date, underwhelming (Melov et al., 2007; Phillips et al., 2013), and we also noted limited overlap between the HypAt-regulated genes and age- or insulin-sensitivity-regulated genes (Figure 5A; Data S1). However, at a pathway level, the overlap between our gene signature and these processes was considerable (Figure 5B). Specific hypotheses can be generated by overlaying the information of transcript signatures for each condition, using tissue-specific network analysis. Annotating quantitative networks in this way revealed various single-gene interactions between exercise, aging, and insulin signaling. For example, we show in Figure 6 an “age network” formed around *LAMTOR5*, a protein subunit of the pentameric Ragulator complex that tethers the Rag GTPases and, by extension, mTORC1, to the lysosomal membrane (Bar-Peled et al., 2012; Sancak et al., 2010). The expression of *LAMTOR5* was not regulated by exercise, yet Figure 6 reveals parts of the *LAMTOR*5 “interactome” relate to muscle activity (e.g., *PRDX1* interacts with *TXNL1*, and *MTRF1* interacts with *LAMTOR5*). These interactions can be explored further using reverse genetic strategies and pharmacological tools in human primary cells (Crossland et al., 2017a).

Any RNA signature can be used to calculate a global ”gene score” per sample (Sood et al., 2015; Timmons et al., 2010), and related to features of aging or frailty (i.e., used as a potential diagnostic) or the gene list can be used to direct targeted DNA sequencing. For example, we identified 141 gene expression responses that quantitatively related to the magnitude of muscle growth (Figure S3, see Table S2 for instructions on how to produce a 3D representation of this network). Several genes have already been extensively validated as regulators of growth and protein synthesis (e.g., *FOXO*, *MAPK*, *AMPK*, *PI3K-Akt*, and *APLNR*) (Ellis et al., 2014; Li et al., 2012; Sandri et al., 2013; Vinel et al., 2018), and we validated four additional genes (see above). There was no statistically significant overlap with the *in vitro* transcriptional signature for rapamycin in primary muscle cells (Timmons et al., 2019) and a rather limited overlap with the mTOR canonical pathway. However, when using the 141-gene signature to interrogate the L1000 drug signature database (Keenan et al., 2018), we did identify that a number of mTOR inhibitors could perturb the 141-gene signature. Unlike human muscle aging (Timmons et al., 2019), this list did not include rapamycin. The cMAP L1000 database also includes transcriptional signatures following gene KD or overexpression (OE), in 8 independent cell lines (Data S1). Overall, we identified 119 drugs that could either mimic or oppose the *in vivo* lean mass 141-gene signature (Data S1), including numerous cyclin-dependent kinase (CDK) inhibitors, which is consistent with our independent primary cell culture experiments, where we found that eEF2 Thr56 phosphorylation status was influenced by *BCAT2* and *FKBP1A* expression. Future studies examining the potential of some of these 119 drugs to interact with exercise-induced muscle adaptation will shed light on whether the matching of transcriptional signatures is predictive of positive synergies or potential aggravation of age-related muscle frailty.

### Limitations of Study

The present study lacked a parallel proteome analysis. To overcome this limitation, we used large-scale protein-protein interaction datasets to establish that our data generate significant networks containing proteins that belong to pathways known to regulate cell growth and protein synthesis. This allowed us to demonstrate strong agreement between our transcriptional approach and the proteome changes identified in a recent publication (Robinson et al., 2017). While our paired loading and UL modeling strategy recapitulated well-known RNA and proteome signatures, we only included men in the HypAt study. Women have shown, compared to men, a differing abundance of genes involved in fatty acid oxidation (Liu et al., 2010); perhaps this is of limited concern, given that in a large (n = 178) analysis of muscle sex differences in physiologically matched subjects, only ∼200 genes are differentially expressed (>1.2 FC, FDR < 1%), and only 2, *ATRNL1* and *TSPYL2*, are in the present analysis. Finally, our method identifies alterations in UTR signal, but it does not catalog the specific details, which can include losses and gains in microRNA (miRNA) target sites, mRNA stability, and alterations in transcript polyadenylation. For example, the increase in *FKBP1A* 3′ UTR signal reflects the increased production of up to 3 transcripts with the long 3′ UTR, each coding for ∼100 amino acid protein.

### Conclusion

We developed a powerful model and analytical tool to measure the within-individual paired tissue responses to loading and UL in human muscle tissue that reduces response heterogeneity by ∼40%, meaning that lower sample sizes can be used to discover insightful transcriptional signatures (MacInnis et al., 2017) before independent validation. Using an innovative genome-wide analysis strategy, we also demonstrated significant regulation of gene expression occurring only at the UTRs, thus providing some insight into the reported discordance between mRNA and corresponding protein levels. Moreover, we discovered that many of these UTR regulatory events covary with lean mass transitions in humans. The global pattern of gene expression was not mTOR dominant; however, we demonstrate some level of mTOR involvement occurring at the protein phosphorylation level. The extensive independent validation and close agreement with previous mechanistic studies supports the use of the HypAt model as a powerful tool to identify causal molecular determinants of muscle growth and atrophy directly in humans using a variety of molecular techniques and interventions.

## STAR★Methods

### Key Resources Table

**Table:** 

REAGENT or RESOURCE	SOURCE	IDENTIFIER
**Antibodies**		

Phospho-mTOR (Ser2448) (D9C2) XP® Rabbit mAb	Cell Signaling Technology	CAT: #5536; RRID: AB_10691552
Phospho-p70 S6 Kinase (Thr389) (108D2) Rabbit mAb	Cell Signaling Technology	CAT: #9234; RRID: AB_2269803
Phospho-eEF2 (Thr56) Antibody	Cell Signaling Technology	CAT: #2331; RRID:AB_10015204
Phospho-4E-BP1 (Thr37/46) (236B4) Rabbit mAb	Cell Signaling Technology	CAT: #2855; RRID:AB_10695878
Anti-Puromycin Antibody, clone 12D10	Merck Millipore	CAT: MABE343; RRID:AB_2566826

**Biological Samples**		

Human Muscle	Present article	NA
Human Muscle	Morton et al., 2016; Mitchell et al., 2014; Phillips et al., 2017	NA

**Chemicals, Peptides, and Recombinant Proteins**		

Deuterium oxide (D, 70%)	Cambridge Isotope Laboratories, Inc.	DLM-4-70-PK; CAS#7732-18-5
Dowex – 50WX8 – Hydrogen Form	Sigma Aldrich	AC335335000
PhosStop Phosphatase Inhibitor	Roche	04906837001
cOmplete Mini EDTA-free Protease Inhibitor Cocktail	Roche	11836170001
TRIzol Reagent	ThermoFisher Scientific	CAT: 15596018
CD56 Microbeads, human	Miltenyi Biotec	CAT: 130-097-042
Lipofectamine RNAiMAX Transfection Reagent	ThermoFisher Scientific	CAT: 13778030
LONG® R3 IGF-I human	Sigma-Aldrich	CAT: I1271
Puromycin dihydrochloride	Sigma-Aldrich	CAT: P8833
SYBR Select Master Mix	Applied Biosystems	CAT: 4472920

**Critical Commercial Assays**		

E.Z.N.A Total RNA Isolation Kit	Omega Bio-Tek	SKU: R6834-01
GeneChip WT Plus Reagent Kit	ThermoFisher Scientific	CAT: 902280
High-Capacity cDNA Reverse Transcription Kit	Applied Biosystems	CAT: 4368813

**Deposited Data**		

Raw and analyzed data	This study	GEO: GSE154846

**Oligonucleotides**		

esiRNA human BCAT2 (esiRNA1)	Sigma-Aldrich	CAT: EHU032991
esiRNA human NID2 (esiRNA1)	Sigma-Aldrich	CAT: EHU083781
esiRNA human FKBP1A (esiRNA1)	Sigma-Aldrich	CAT: EHU106961
esiRNA human MBNL1 (esiRNA1)	Sigma-Aldrich	CAT: EHU086561
Primer: BCAT2 Forward: GAGCTGAAGGAGATCCAGTACG	Sigma-Aldrich	N/A
Primer: BCAT2 Reverse: GAGTCATTGGTAGGGAGGCG	Sigma-Aldrich	N/A
Primer: NID2 Forward: TGGAAGCTACAGGTGTGAGTG	Sigma-Aldrich	N/A
Primer: NID2 Reverse: AGGTGGGGTGATCAAGATGCAA	Sigma-Aldrich	N/A
Primer: MBNL1 Forward: CTGCCCAATACCAGGTCAAC	Sigma-Aldrich	N/A
Primer: MBNL1 Reverse: GGGGAAGTACAGCTTGAGGA	Sigma-Aldrich	N/A
Primer: FKBP1A Forward: GGTGGAAACCATCTCCCCAG	Sigma-Aldrich	N/A
Primer: FKBP1A Reverse: TCAAGCATCCCGGTGTAGTG	Sigma-Aldrich	N/A

**Software and Algorithms**		

ImageJ	Schneider et al., 2012	https://imagej.nih.gov
Bowtie Alignment Tool	Langmead and Salzberg, 2012	http://bowtie-bio.sourceforge.net/index.shtml
Significance Analysis of Microarrays (SAM) implemented in the R programming environment (SAMR)	Tusher et al., 2001	https://cran.r-project.org/web/packages/samr/index.html
aroma.affymetrix	Bengtsson et al., 2008	https://cran.rstudio.com/web/packages/aroma.affymetrix/index.html
MEGENA	Song and Zhang, 2015	https://rdrr.io/cran/MEGENA/
Bioconductor	Gentleman et al., 2004	https://www.bioconductor.org/
CMap-L1000v1 database	Subramanian et al., 2017	https://clue.io/
Custom CDF protocol	Timmons et al., 2018	https://www.augurprecisionmedicine.com
Ciiider	Gearing et al., 2019	www.ciiider.org

**Other**		

GeneChip Human Transcriptome Array 2.0 and RT labeling kits	ThermoFisher Scientific	CAT: 902162
Raw and analyzed data	Timmons et al., 2018, 2019	GEO: GSE154846
Human reference genome NCBI build 38, GRCh38_82p3	Genome Reference Consortium	https://www.ncbi.nlm.nih.gov/projects/genome/assembly/grc/human/

### Resource Availability

#### Lead Contact

Further information and requests for resources should be directed to and will be fulfilled by the Lead Contact, Dr. Stuart M. Phillips (phillis@mcmaster.ca)

#### Materials Availability

This study did not generate any unique reagents.

#### Data and Code Availability

The accession number for the newly generated gene expression data reported in this paper is GEO:GSE154846. Code for the various informatics analyses can be readily obtained by contacting jamie.timmons@gmail.com.

### Experimental Model and Subject Details

#### Human Participants

Participants were recruited via local advertisements posted at McMaster University. Twelve recreationally-active men (18-30 y) volunteered to participate in the study. A medical history questionnaire was administered at screening to exclude any individuals taking medications known to affect protein metabolism (i.e., glucocorticoids, prescription-strength acne medication, or non-steroidal anti-inflammatories), those with a (family) history of deep vein thrombosis, or individuals with acute or chronic illnesses that interfered with the safe conduct of the study. All participants provided informed verbal and written consent prior to beginning the study. The experimental trial was approved by the Hamilton integrated Research Ethics Board (REB #2867) and complied with the ethical standards outlined in the Tri-Council Policy statement for use of humans in research. This study was registered on CinicalTrials.gov with the identifier NCT03046095.

#### Human Primary Muscle Cell Culture

Human primary skeletal muscle cells were isolated from muscle biopsies from healthy young adults and cultured as previously described (Crossland et al., 2017a, 2017b). Myogenic cell enrichment was carried out using magnetic-activated cell sorting (MACS), using anti-CD56 microbeads and myoblasts were used for experimentation at passage 5-6. MACS-sorted myoblasts were cultured on Collagen Type I-coated 6-well dishes in Dulbecco’s Modified Eagle Medium/Nutrient Mixture F-12 (DMEM/F-12; Life Technologies) containing 20% (v/v) fetal bovine serum (FBS, Sigma-Aldrich), 1% (v/v) antibiotic-antimycotic (AbAm) solution and 4mM L-glutamine (Life Technologies). Once cells reached ∼95% confluency, differentiation was induced by switching the medium to DMEM/F-12 containing 2% (v/v) horse serum (Sigma-Aldrich), 4mM L-glutamine and 1% (v/v) AbAm solution (Life Technologies). Puromycin based measures of protein synthesis was determined as previously described (Crossland et al., 2017b).

### Method Details

#### Experimental Protocol

The total duration of the study, including familiarization sessions, was 11 weeks. We employed a within-subjects design whereby one leg was randomized to perform unilateral leg extension and leg press exercise 3 d·wk^-1^ for 10 weeks. The contralateral leg had a knee brace applied during the last two weeks of the study, and otherwise remained untrained. Body composition and maximal strength assessments were performed before training (Pre-RT; day 0), before immobilization (Pre-UL; day 56), and upon completion of training and immobilization (Post-RT/UL; day 70). At each testing session, participants had their lean body mass (LBM), mid-thigh vastus lateralis cross sectional area (VLCSA), and ISO-MVC assessed. VLCSA, muscle volume, and whole-quadriceps CSA was quantified using magnetic resonance imaging (MRI) at time points Pre-RT and Post-RT. A total of 7 muscle biopsies were obtained from the *vastus lateralis* throughout and used, in conjunction with deuterium oxide, to measure integrated rates of muscle protein synthesis (see below).

#### Pretesting

Participants visited McMaster University on three separate occasions at least one week prior to beginning the study to be familiarized on the proper execution of an isometric maximal voluntary contraction (ISO-MVC) and unilateral leg extension and leg press exercises. One-repetition maximum (1-RM) was tested on visits 2 and 3. Proper execution of unilateral leg extension and leg press contractions was demonstrated, after which participants performed one set of non-exhaustive contractions while having their form critiqued and adjusted. Participants then completed 3 sets of leg extension followed by 3 sets of leg press, with each set separated by a 90 s rest period. On the final set of each exercise, participants were instructed to reach volitional failure, defined as an inability to complete a full contraction through the predefined range of motion. Weight lifted, and the number of repetitions completed for both leg extension and leg press exercises, was recorded and used to calculate estimated 1-RM using the following formula:

(Equation 1) 1−RM(kg)=(Load(kg))/(1.0278-(0.0278x#reps))

On the second visit (∼48hr after visit 1), participants completed two submaximal sets of 8-12 repetitions on the leg extension machine at ∼40%–60% of their estimated 1-RM. Participants then attempted to lift 90% of their 1-RM predicted from Equation 1. Weight was increased progressively until the participant could not complete a full repetition. This testing procedure was repeated on the leg press after ∼10 mins of rest. On the third visit,1-RM load was retested and either verified or adjusted accordingly and used for the subsequent calculation of week 1 working loads.

#### Dietary Records and Activity Monitoring

Participants completed 3-d dietary records during week 1, week 5 and week 10 of the study. Each record consisted of two weekdays and one weekend day. A sample record was provided to each participant to permit the accurate estimation of portion sizes. Dietary records were analyzed using NutriBase software (Cybersoft Inc., version 11.5, Phoenix, AZ, USA). During dietary recording, participants were also asked to wear an ActiGraph wGT3X-BT activity monitor (ActiGraph, Pensacola, FL, USA) on their dominant wrist to track daily step count and other metrics of physical activity levels. Data were downloaded from the activity monitors and analyzed using ActiLife version 6 13.2 software (ActiGraph, Pensacola, FL, USA).

#### Unilateral Resistance Exercise - HypAt

Participants visited the laboratory every Monday, Wednesday, and Friday to complete unilateral leg extension and leg press exercise. Each session was supervised by a strength and conditioning coach and consisted of 3 sets of 8-12 repetitions of leg extensions and 3 sets of 8-12 repetitions on a 45-degree leg press. The last set of each exercise was performed to volitional failure, defined as an inability to complete a repetition through the full range of motion. If the participant successfully completed more than 12 or less than 8 repetitions, weight was adjusted accordingly. Following each exercise bout, participants ingested 25 g of whey protein isolate to maximize the protein synthesis response to each exercise bout.

#### Immobilization

A X-ACT ROM knee brace (DonJoy, Dallas, TX, USA) was applied to the contralateral non-training leg for a continuous period of 14 d during weeks 9 and 10. The angle of the brace was adjusted to permit toe clearance during crutch-assisted ambulation without active hamstring flexion and averaged ∼60° flexion.

#### Dual energy X-ray absorptiometry

Leg lean mass was assessed using dual energy X-ray absorptiometry Pre-RT, Pre-UL, and Post-RT/UL. The DXA was calibrated daily prior to participant arrival using a three-compartment Universal Whole Body DXA Phantom (Orthometrix, Naples, Florida). Participants also underwent a fasted MRI scan of both thighs on day 0 and upon study completion (day 70) for the assessment of mid-thigh and whole-quadriceps CSA (see below).

#### Muscle Strength

Isometric maximal voluntary contractions (ISO-MVC) were performed prior to exercise Pre-RT, Pre-UL, and after exercise completion to assess peak knee extensor torque using a Biodex dynamometer (Biodex System 3, Biodex Medical Systems, Shirley, NY, USA). Participants were familiarized on three separate occasions during the pretesting visits to avoid practice effects associated with device familiarity. During the pre-test familiarization sessions, chair settings were adjusted to each participant such that the knee joint was aligned with the axis of rotation of the machine. All chair settings were recorded for accurate replication during subsequent testing sessions. Each session consisted of five isometric contractions at 60° from the resting, 90° neutral position. Contractions lasted 5 s and were separated by a 2-min resting period. Both legs were tested at each session in randomized order. Participants were shown their force tracing and verbally encouraged during each effort to limit the influence of motivation on trial performance. Peak torque was recorded and used in subsequent analyses. The coefficient of variation between contractions did not exceed 5%.

#### Deuterium Oxide Protocol

The incorporation of deuterium oxide (D2O) into muscle protein-bound alanine was assessed to quantify muscle protein synthesis rates. Deuterium dosing began on day 52 and continued until day 70. The protocol consisted of one loading day and 17 maintenance days with the goal of enriching, and subsequently maintaining the body water pool at ∼0.5% 2H atom percent excess (APE). Participants reported to the laboratory at 0800 h after an overnight (∼10 h) fast on day 52 (Thursday) and were asked to void their bladder before undergoing a DXA body scan to determine lean body mass. Participants then ingested 8 doses (0.625 ml·kg-1 LBM) of 70% D2O (Cambridge Isotope Laboratories, Andover, MA, USA) evenly spaced every 1.5 h throughout the day. After 5 of the 8 doses were ingested, participants were sent home with the remaining 3 doses and instructed to ingest them 1.5 h apart. None of the participants reported any adverse side effects such as nausea or vertigo after consuming D2O. Blood samples were collected in EDTA blood tubes and saliva samples were obtained by gently chewing on a cotton swab for 2-3 min until completely saturated with saliva. Blood and saliva samples were centrifuged at 4000 rpm for 10 min at 4°C, after which aliquots of each were snap frozen in liquid nitrogen and stored at −80°C for subsequent analysis.

#### Saliva Analysis

Saliva samples were obtained at baseline prior to commencing deuterium oxide loading on day 52 and each morning thereafter until the end of the study. Salivettes were centrifuged at 4000 rpm for 10 min and diluted in doubly-distilled water. Deuterium enrichment was then measured using a Picarro L2130-*i* Cavity Ringdown Spectrometer run in high-throughput mode. Six injections were performed on each sample; the first readings were discarded to eliminate memory effects from previous sample injections. Internal lab standards containing low, medium, and high enrichments of ^2^H were sampled in parallel prior to and following each participant’s samples to account for drift in enrichment over time.

#### Magnetic Resonance Imaging

Participants underwent a fasted MRI scan of both thighs on day 0 and upon study completion (day 70) for the assessment of mid-thigh and whole-quadriceps CSA. Each scan was performed in a 3-Tesla HD Scanner (Signa MRI System; GE Medical, Milwaukee, Wisconsin) at the Imaging Research Center (St. Joseph’s Healthcare, Hamilton, ON). Axial (transverse) MR images were obtained from both thighs from the distal end of the femur to greater trochanter. A fast-recovery, fast spin echo (FRFSE) pulse sequence was used, along with IDEAL (iterative decomposition of water and fat with echo asymmetry and least-squares estimation) post-processing to obtain water-only, fat-only, in-phase and out-of-phase images of the thighs. The following parameters were used: (TR) = 2000 msec, (TE) = 30 msec, refocusing flip angle = 111 degrees, echo train length = 6, ASSET (parallel imaging factor) = 2, field of view = 42x21 cm, acquisition matrix = 512x256, 3 mm slice thickness, and 0mm slice gap. A total of ∼160 slices were acquired from each participant. The acquisition was completed in two sections: a lower stage and an upper stage, which were subsequently stitched together by an MR technician. Total scan time for both stages was approximately 11 min. Images were downloaded from a secure server and analyzed using ImageJ (NIH, v 1.52). Mid-thigh vastus lateralis CSA was analyzed at 50% of the distance between the greater trochanter and lateral epicondyle of the femur. Peak-quadriceps CSA was the slice with the greatest measured quadriceps CSA. Finally, quadriceps muscle volume was calculated by summing the CSA measurements of a given slice multiplied by the slice thickness. Peak-quadriceps CSA and muscle volume were analyzed using semi-automatic image analysis software (AnalysisPro). The distal 20% of thigh images and the proximal 30% were not included in analyses. This data was not utilized as the additional three clinical studies only had DXA measures of muscle mass.

#### Muscle Tissue Extraction

Muscle biopsy samples (∼100 – 150 mg each) were obtained on 7 occasions under local anesthesia using a Bergstrom needle modified for manual suction. Biopsies were taken from the RT limb pre-RT (day 0), day 53, day 56 and day 70 and from the UL limb on day 53, 56, and 70. Blood and other non-muscle tissue were dissected from each specimen at bedside prior to being snap-frozen in liquid nitrogen (within ∼20 s) and stored at −80°C.

#### RNA Extraction and Transcriptome Profiling

Approximately 20 mg of muscle was used to extract RNA. Muscle samples and 1000 μL of TRIzol were added to Lysing Matrix D tubes containing ceramic microbeads (MP Biomedicals, Solon, OH, USA) and homogenized using a FastPrep tissue homogenizer (MP Biomedicals, Solon, OH, USA). 200 μL of chloroform was added and the tubes hand-shaken vigorously for 15 s and incubated at room temperature for 5 min. Samples were then centrifuged at 12 000 g for 10 min at 4°C and the upper aqueous phase containing RNA was transferred to an RNase-free tube. RNA was purified using E.Z.N.A Total RNA Isolation kit (Omega Bio-Tek, Norcross, GA, USA). RNA was processed for transcriptome profiling using the GeneChip WT Plus Reagent Kit according to manufacturer’s instructions. Briefly, first and second strand cDNA synthesis was performed using 100 ng of RNA and a spike-in Poly-A control, which was then followed by reverse transcription into cRNA. cRNA was purified using magnetic beads and quantified using spectrophotometry (Nanodrop UV-Vis, Thermo Fisher Scientific). 15 μg of cRNA was then amplified and hydrolyzed using RNase H (leaving single-stranded cDNA) and purified with magnetic beads. cDNA (5.5 μg) was then fragmented and labeled. A hybridization master mix was prepared and added to the fragmented and labeled cDNA. 200 μL of the mixture was applied to the HTA 2.0 cartridge and hybridized at 45°C for 16 h rotating at 60 rpm. The cartridge was washed and stained using the FS450_001 fluidics protocol on the GeneChip Fluidics Station 450 (Thermo Fisher) and scanned using a GeneChip Scanner 3000 7G (Thermo Fisher).

#### Myofibrillar Extraction

Snap-frozen muscle samples were homogenized using buffer (10 μl·mg^-1^) containing 25 mM Tris buffer (pH 7.2), 0.5% vol/vol Triton X-100, a phosphatase inhibitor (PhosStop) and a complete protease inhibitor tablet (Roche, Mississauga, ON, CA). Samples were centrifuged at 4500 rpm for 10 min at 4°C and the supernatant was removed. Collagen was precipitated with 1M NaOH and discarded leaving a myofibrillar-enriched supernatant fraction. Perchloric acid (1M) was added to the supernatant to precipitate the myofibrillar fraction. Proteins were then hydrolyzed by adding 1 mL of Dowex resin (50WX8-200) and 1 mL of 1M HCl to each sample followed by subsequent incubation for 72 hr at 110°C. Samples were vortexed every 24 hr. Free amino acids were then isolated using Dowex ion-exchange chromatography and the N-acetyl-*n-*propyl ester of alanine was prepared and analyzed by gas chromatography pyrolysis isotope ratio mass spectrometry.

#### Calculations

##### Saliva

Saliva deuterium enrichments were provided as a ratio (δ^2^H), where:

(Equation 2) $$\delta^{2}Η\;=\left(\;\frac{\left(2H/1H\right)\;sample}{\left(2H/1H\right)\;standard}-1\right)*1000$$

Atom percent values were calculated as previously described:

(Equation 3) $$AtomPercent\;=\frac{100\text{ x AR x }\left(\text{δ}2\text{H x }0.001+1\right)}{1+\text{AR}\left(\text{δ}2\text{H x }0.001+1\right)}$$

Where AR is the absolute ratio constant for deuterium (0.00015595) based on the VSMOW standard. Atom percent excess was calculated by subtracting background deuterium enrichment (at time = 0) from each sample and multiplying the result by 35 to account for sample dilution.

##### Integrated Muscle Protein Synthesis Rates

Myofibrillar protein synthesis was calculated using the standard precursor-product equation:

(Equation 4) $$\;\;iMyoPS\;\left(\%\cdot d^{-1}\right)=\left[\frac{\left(\Delta \;APE_{Ala\;}\right)\;}{APE_{BW}\;\times \;3.7\;x\;\;t\;}\right]\;\times \;100$$

Where $\Delta \;APE_{Ala\;}$is the change in protein-bound ^2^H-alanine enrichment between biopsy sampling points, $APE_{BW}$ is the body-water ^2^H enrichment multiplied by 3.7 to correct for the number of carbon-hydrogen bonds labeled in alanine relative to total body water, and *t* is the incorporation time (in days) between biopsies. RT-induced change in iMyoPS was calculated using the muscle deuterium enrichment values calculated in the muscle specimen taken from the RT limb on day 70 and the UL limb on day 56 (i.e., rested limb). The effect of UL on iMyoPS was calculated using the muscle deuterium enrichment values calculated in the muscle specimens taken from the UL limb on day 70 and from the UL limb on day 56.

#### Exercise Protocols used in Independent Studies

##### Morton et al. (2016)

For a detailed description of the RT protocol used, refer to Morton et al. (2016). Participants were 23 ± 2 years old, with a BMI of 26.9 ± 2 kg/m^2^ and had 4-5 y of resistance training experience. Briefly, participants performed full-body resistance training 4 days/week (Mon, Tues, Thurs, Fri) for 12 weeks that targeted all of the major muscle groups. Each session included 5 exercises, each of which were performed for 3 sets to volitional failure. Participants were randomly assigned to perform either high-repetition (20-25 reps per set @ 30%–50% 1-RM) or low-repetition training (8-12 reps per set @ 75%–90% 1-RM). Indices of muscle hypertrophy did not significantly differ between the groups post-RT, so group allocation was not considered in the present use of muscle tissue samples. Participants consumed 30 g of protein after each exercise bout. Muscle biopsies were taken from the *vastus lateralis* using the Bergstrom approach described above prior initiating the RT protocol and 72 h after the final RE bout at 12 weeks. RNA was extracted from whole muscle samples and analyzed on the HTA 2.0 platform using an identical method as described above (see RNA Extraction and Transcriptome Profiling).

##### Mitchell et al. (2014)

For a detailed description of the protocol used, refer to Mitchell et al. (2014). Participants were 24 ± 1 years old, with a BMI of 26.4 kg/m^2^ and were recreationally active (not participating in an RT program). Participants performed full-body resistance training 4 days/week for 16 weeks consisting of two upper body and two lower body training sessions per week. The program progressed from 3 sets of 12 repetitions to 4 sets of 6 repetitions of each exercise. The last set of each exercise was performed to volitional failure. Participants consumed 30 g of protein immediately after each exercise session. Biopsies were obtained from the *vastus lateralis* using the Bergstrom approach described above prior to initiating 16 weeks of RT and ∼48-72 h following the last RE bout. RNA was extracted from whole muscle samples as described above (see RNA Extraction and Transcriptome Profiling). Samples were dissolved in RNase-free water, processed to single-stranded sense fragmented DNA using the GeneChip® WT PLUS Reagent Kit, which relies on a reverse transcription priming strategy that primes both the poly-A and non-poly-A RNA. HTA 2.0 chips were processed according to the manufacturer’s protocol. Fragmented (5 μg) end-labeled sense strand target cDNA was hybridized to each array and scanned using a Gene Chip Scanner 30007G (Affymetrix Core, MPI A/S, Denmark).

##### Phillips et al. (2017)

For a detailed description of the protocol used, refer to Phillips et al. (2017). Participants performed high-intensity interval training (HIIT) 3 days/week (Mon, Wed, and Fri) for 6 weeks. Each session consisted of a 2 min warmup at 50 W followed by 5 sets of high intensity cycling at ∼125% VO_2_max for 1 minute. Each set was separated by 90 s of rest. Biopsy tissue was available at pre and post time points for 47 participants, including 16 males and 31 females with an average age of 39 years (range 20-51 years) and a BMI of 31 kg/m^2^ (27-43 kg/m^2^). RNA was extracted using TRizol® (Life Technologies), dissolved in RNase-free water, processed to single-stranded sense fragmented DNA using the GeneChip® WT PLUS Reagent Kit, which relies on a reverse transcription priming strategy that primes both the poly-A and non-poly-A RNA. HTA 2.0 chips were processed according to the manufacturer’s protocol. Fragmented (5 μg) end-labeled sense strand target cDNA was hybridized to each array and scanned using a Gene Chip Scanner 30007G (Affymetrix Core, MPI A/S, Denmark).

#### siRNA Transfection Experiments

Four days following initiation of myocyte differentiation, siRNA transfection was carried out following a media change, using lipofectamine RNAiMAX (Invitrogen), according to the manufacturer’s instructions. Gene-specific knockdown was achieved using 500ng human Mission® esiRNA (Sigma-Aldrich) for the following genes: BCAT2 (EHU032991), NID2 (EHU083781), FKBP1A (EHU106961) and MBNL1 (EHU086561). Thirty-six hours following transfection, a media change was carried out and long R3 IGF-1 (50 ng/ml; Sigma-Aldrich) was added to each well. Cells were harvested after 4h in TRIzol (Life Technologies) for RNA extraction (n = 2), or homogenization buffer (50 mM Tris-HCl, pH7.5, 1 mM EDTA, 1 mM EGTA, 10 mM β-glycerophosphate, 50 mM NaF and complete protease inhibitor cocktail tablet (Roche, West Sussex, UK)) for protein extraction (n = 4). A subset of cells was also collected after 48h in 0.3 mol/l NaOH for total protein, RNA and DNA extraction and quantification (n = 5). For 4h experiments, 0.5 μM puromycin (Sigma-Aldrich, UK) was added to each well at the point of IGF-1 addition.

#### Cell culture total RNA Extraction and RT-PCR

Total RNA was extracted using TRIzol (Life Technologies) according to the manufacturer’s protocol. RNA was resuspended in 20 μl of RNase-free water and quantified using a NanoDrop (Thermo Scientific). cDNA was synthesized by reverse transcription using the High Capacity cDNA synthesis kit (Applied Biosystems) with 500 ng RNA. Samples were subsequently diluted 1:5 using RNase-free water and real-time PCR was performed using 1 μl cDNA in duplicate and 6 μl master mix containing SYBR Select Master Mix (Life Technologies) with primers targeting the following genes: BCAT2 (Fwd: GAGCTGAAGGAGATCCAGTACG, Rev: GAGTCATTGGTAGGGAGGCG), NID2 (Fwd: TGGAAGCTACAGGTGTGAGTG, Rev: AGGTGGGGTGATCAAGATGCAA), MBNL1 (Fwd: CTGCCCAATACCAGGTCAAC, Rev: GGGGAAGTACAGCTTGAGGA), and FKBP1A (Fwd: GGTGGAAACCATCTCCCCAG, Rev: TCAAGCATCCCGGTGTAGTG). RPL13A was used for as a housekeeping gene since it was stable between each group. PCR was performed using a Viia 7 real-time PCR machine (Life Technologies) using the following thermal cycling conditions: 2 min at 50°C, 10 min at 95°C and 40 cycles of 15 s at 95oC and 1 min at 60°C.

#### Cell culture Western Blotting

Protein extraction from cells was performed by repeatedly passing samples through gel-loading pipette tips. Samples were centrifuged at 13,000 g for 10 min at 4°C. Protein samples (5 μg) were loaded onto Criterion XT 12% Bis-Tris gels (Bio-Rad) at 200V for 1h, then transferred to PVDF membrane for 45 min at 100V. After this, membranes were blocked using 5% (w/v) milk for 1h at room temperature, then incubated with primary antibodies all diluted 1:2000 (phosphorylated mTOR Ser2448 (#5536), phosphorylated p70 S6K1 Thr389 (#9234), phosphorylated eEF2 Thr56 (#2331), and phosphorylated 4E-BP1 Thr37/46 (#2855); all from Cell Signaling Technology), except for anti-puromycin (Millipore), which was diluted 1:5000. Membranes were incubated overnight at 4°C. The following day, membranes were washed 3x5 min with 1x TBS-Tween, then incubated with HRP-conjugated anti-rabbit secondary antibody (New England) 1:2000 for 1h at room temperature. Bands were detected by incubating with enhanced chemiluminescence detection reagent (Millipore) and exposing in a Chemidoc XRS system (Bio-Rad). Bands were normalized against GAPDH levels. For total Protein, RNA and DNA measurements (48h) cells were harvested in 0.3 mol/L NaOH and then incubated at 37°C for 20 minutes (extraction of total alkaline-soluble protein). After protein quantification, 1 mol/L PCA was added to each sample, which were then left at 4oC for 30 minutes. After centrifugation, the supernatant was quantified for RNA. Finally, to the pellet, 2 mol/L PCA was added and samples were incubated at 70°C for 1h. The resultant supernatant was used to quantify DNA.

### Quantification and Statistical Analyses

#### Physiological Data

Physiological data were assessed for normality using the Shapiro-Wilk test and visually inspected using Q-Q plots. Paired-samples t tests were conducted to test for baseline differences in leg lean mass, mid-thigh CSA, and ISO-MVC between the trained and immobilized legs. Changes in physical activity and macronutrient intake over the duration of the study were analyzed using a one-way repeated-measures ANOVA. Changes in ISO-MVC, mid-thigh CSA and body composition variables obtained via DEXA were analyzed using a two-way repeated-measures analysis of variance (ANOVA) with time (2) and leg (2) as the within-subjects’ factors. Muscle protein synthesis was analyzed using a one-way ANOVA with repeated-measures. In all cases, the statistical procedures were conducted on 12 young men (n). Results are presented as mean response plus/minus SD and, where appropriate, the median and range is included to emphasize variation in response. A Tukey’s HSD post hoc test was employed to probe for pairwise differences between legs and across time points where warranted. In all analyses, statistical significance was set at p ≤ 0.05.

#### Transcriptomic Data

All of the new HTA 2.0 array data has been deposited along with existing array data and are available at GEO (GSE154846). Standard quality control processes were performed for each study (NUSE plots and PCA). While the array offers advantages in terms of reproducibility, standard pre-processing approaches do not account for study specific probe-performance, and we have developed a study-specific pipeline that scans the 7 million short probes prior to assembly of transcripts using the following criteria:

a) specific to one location on the genome
b) the probe signal is above background noise in the particular dataset

Data analysis methods utilized numerous informatics resources (Bengtsson et al., 2008; Dai et al., 2005; Gentleman et al., 2004; Wang et al., 2012). Each transcriptional ‘unit’ of expression was defined by establishing which of the ∼6.9 million probes, from the HTA 2.0 chip, were detectable, and then assembling the signal from GC corrected ‘active’ probes into probe-sets (defined by ensembl ENST identifiers, http://www.ensembl.org//useast.ensembl.org/?redirectsrc=//www.ensembl.org%2F). The custom chip definition file (CDF) used to summarize the transcript level data is deposited at GEO. Our method provides an enhanced signal by removing individual probes, from probe-sets, that approximate to background noise. To create each CDF, the ∼6.9 million probe sequences (Affymetrix website) were aligned to the genome build e.g., GRCh38_82p3 (e.g., see http://brainarray.mbni.med.umich.edu for a description) using bowtie alignment tool (Langmead and Salzberg, 2012). Probes which map to more than one part of the genome are discarded. There are ∼50,000 probes on the HTA 2.0 array which have an extreme GC content (i.e., < 20%, > 80%) and these were removed as the adjustment model used to correct GC content is not effective at these extreme values. Older-generation transcriptomic data was re-processed to use the same transcript identifiers from the updated HTA pipeline (Timmons et al., 2018).

Most studies of human muscle to date have used RNA detection technology that is unable to accurately measure exon-specific transcripts (Shalek et al., 2013) or provides partial and skewed coverage of the transcriptome. Our process allows us to define which part of the transcript we wish to quantify and in the present study, we apply the custom CDF approach to study differential expression of specific regions of transcripts through comparing the differential expression (DE) response of the entire (‘full length’) ensembl defined transcript unit, with the responses of only the untranslated regions – regions critical for regulating protein translational efficiency and hence a key component of the cell hypertrophy response. For DE analysis, we utilized paired SAMR analysis using a cut off of 5% FDR and 1.2-fold difference to generate the initial transcript lists - before applying a number of down-stream approaches to subset the data (See Results). For network analysis, we used the R-package MEGENA (Song and Zhang, 2015) to identify network structures (FDR < 1% for spearman correlation; p < 0.01 for module significance and p < 0.01 for network connectivity) and 10,000 permutations for calculating FDR and connectivity p values. Network data-plots were produced using Fruchterman-Reingold force directed plotting within MEGENA (Song and Zhang, 2015). CiiiDER was used to identify potential regulatory transcription factors (Gearing et al., 2019).

To estimate if a UTR signal was regulated in a manner distinct from the full length (FL) ENST’s the following heuristic was utilized:

1) A “gene” was considered if it was significantly regulated in any one of the three statistical analysis comparisons between RT and UL (FL-ENST, 3′UTR and 5′UTR values, FDR < 5%, > 1.2FC)

AND

2) the FL-ENST was neither statistically regulated, nor the absolute numerical FC values were > 30% different from the matching *statistically* significant UTR response

OR

3) the FL-ENST was *statistically* regulated but the absolute numerical FC values were > 30% different from the *numerical* UTR response e.g., to include discordant patterns

## Supplemental Information

Document S1. Figures S1–S7 and Tables S1 and S2

Data S1. Differential Gene Expression Data and Downstream Analyses, Related to Figures 1, 3, 5, 6, S2, and S3

Data S2. Input Gene List to Construct 3D Proteome Network, Related to Figures 3 and S3, Table 1, and Table S2

Document S2. Article plus Supplemental Information

## References

[bib1] Abadi A., Glover E.I., Isfort R.J., Raha S., Safdar A., Yasuda N., Kaczor J.J., Melov S., Hubbard A., Qu X. (2009). Limb immobilization induces a coordinate down-regulation of mitochondrial and other metabolic pathways in men and women. PLOS ONE.

[bib2] Adami A., Hobbs B.D., McDonald M.N., Casaburi R., Rossiter H.B., COPDGene Investigators (2018). Genetic variants predicting aerobic capacity response to training are also associated with skeletal muscle oxidative capacity in moderate-to-severe COPD. Physiol. Genomics.

[bib3] Aylett C.H., Sauer E., Imseng S., Boehringer D., Hall M.N., Ban N., Maier T. (2016). Architecture of human mTOR complex 1. Science.

[bib4] Bar-Peled L., Schweitzer L.D., Zoncu R., Sabatini D.M. (2012). Ragulator is a GEF for the rag GTPases that signal amino acid levels to mTORC1. Cell.

[bib5] Bengtsson H., Simpson K., Bullard J., Hansen K. (2008). aroma.affymetrix: a generic framework in R for analyzing small to very large Affymetrix data sets in bounded memory. https://statistics.berkeley.edu/sites/default/files/tech-reports/745.pdf.

[bib82] Brook M.S., Wilkinson D.J., Mitchell W.K., Lund J.N., Phillips B.E., Szewczyk N.J., Greenhaff P.L., Smith K., Atherton P.J. (2016). Synchronous deficits in cumulative muscle protein synthesis and ribosomal biogenesis underlie age-related anabolic resistance to exercise in humans. Journal of Physiology.

[bib6] Camera D.M., Burniston J.G., Pogson M.A., Smiles W.J., Hawley J.A. (2017). Dynamic proteome profiling of individual proteins in human skeletal muscle after a high-fat diet and resistance exercise. FASEB J..

[bib7] Chang J.W., Zhang W., Yeh H.S., de Jong E.P., Jun S., Kim K.H., Bae S.S., Beckman K., Hwang T.H., Kim K.S. (2015). mRNA 3′-UTR shortening is a molecular signature of mTORC1 activation. Nat. Commun..

[bib8] Chang R., Karr J.R., Schadt E.C. (2015). Causal inference in biology networks with integrated belief propagation. Pac. Symp. Biocomput..

[bib9] Chawla R.K., Stackhouse W.J., Wadsworth A.D. (1975). Efficiency of alpha-ketoisocaproic acid as a substitute for leucine in the diet of the growing rat. J. Nutr..

[bib10] Chugunova A., Loseva E., Mazin P., Mitina A., Navalayeu T., Bilan D., Vishnyakova P., Marey M., Golovina A., Serebryakova M. (2019). LINC00116 codes for a mitochondrial peptide linking respiration and lipid metabolism. Proc. Natl. Acad. Sci. USA.

[bib11] Cohain A., Divaraniya A.A., Zhu K., Scarpa J.R., Kasarskis A., Zhu J., Chang R., Dudley J.T., Schadt E.E. (2017). Exploring the reproducibility of probabilistic causal molecular network models. Pac. Symp. Biocomput..

[bib83] Copley Salem C., Ulrich C., Quilici D., Schlauch K., Buxton I.L.O., Burkin H. (2018). Mechanical strain induced phospho-proteomic signaling in uterine smooth muscle cells. Journal of Biomechanics.

[bib12] Crossland H., Atherton P.J., Strömberg A., Gustafsson T., Timmons J.A. (2017). A reverse genetics cell-based evaluation of genes linked to healthy human tissue age. FASEB J..

[bib13] Crossland H., Smith K., Atherton P.J., Wilkinson D.J. (2017). A novel puromycin decorporation method to quantify skeletal muscle protein breakdown: a proof-of-concept study. Biochem. Biophys. Res. Commun..

[bib14] Da Boit M., Sibson R., Meakin J.R., Aspden R.M., Thies F., Mangoni A.A., Gray S.R. (2016). Sex differences in the response to resistance exercise training in older people. Physiol. Rep..

[bib15] Dai M., Wang P., Boyd A.D., Kostov G., Athey B., Jones E.G., Bunney W.E., Myers R.M., Speed T.P., Akil H. (2005). Evolving gene/transcript definitions significantly alter the interpretation of GeneChip data. Nucleic Acids Res..

[bib16] Damas F., Angleri V., Phillips S.M., Witard O.C., Ugrinowitsch C., Santanielo N., Soligon S.D., Costa L.A.R., Lixandrão M.E., Conceição M.S. (2019). Myofibrillar protein synthesis and muscle hypertrophy individualised responses to systematically changing resistance training variables in trained young men. J. Appl. Physiol..

[bib17] Darshi M., Mendiola V.L., Mackey M.R., Murphy A.N., Koller A., Perkins G.A., Ellisman M.H., Taylor S.S. (2011). ChChd3, an inner mitochondrial membrane protein, is essential for maintaining crista integrity and mitochondrial function. J. Biol. Chem..

[bib79] Davidsen P.K., Gallagher I.J., Hartman J.W., Tarnopolsky M.A., Dela F., Helge J.W., Timmons J.A., Phillips S.M. (2011). High responders to resistance exercise training demonstrate differential regulation of skeletal muscle microRNA expression. Journal of Applied Physiology.

[bib18] Diebold L.P., Gil H.J., Gao P., Martinez C.A., Weinberg S.E., Chandel N.S. (2019). Mitochondrial complex III is necessary for endothelial cell proliferation during angiogenesis. Nat. Metab..

[bib19] Ellis B.C., Graham L.D., Molloy P.L. (2014). CRNDE, a long non-coding RNA responsive to insulin/IGF signaling, regulates genes involved in central metabolism. Biochim. Biophys. Acta.

[bib20] Escobar J., Frank J.W., Suryawan A., Nguyen H.V., Van Horn C.G., Hutson S.M., Davis T.A. (2010). Leucine and alpha-ketoisocaproic acid, but not norleucine, stimulate skeletal muscle protein synthesis in neonatal pigs. J. Nutr..

[bib21] Floor S.N., Doudna J.A. (2016). Tunable protein synthesis by transcript isoforms in human cells. eLife.

[bib22] Gearing L.J., Cumming H.E., Chapman R., Finkel A.M., Woodhouse I.B., Luu K., Gould J.A., Forster S.C., Hertzog P.J. (2019). CiiiDER: a tool for predicting and analysing transcription factor binding sites. PLOS ONE.

[bib23] Gentleman R.C., Carey V.J., Bates D.M., Bolstad B., Dettling M., Dudoit S., Ellis B., Gautier L., Ge Y., Gentry J. (2004). Bioconductor: open software development for computational biology and bioinformatics. Genome Biol..

[bib24] Ghadiali R.S., Guimond S.E., Turnbull J.E., Pisconti A. (2017). Dynamic changes in heparan sulfate during muscle differentiation and ageing regulate myoblast cell fate and FGF2 signalling. Matrix Biol..

[bib25] Gibson J.N., Halliday D., Morrison W.L., Stoward P.J., Hornsby G.A., Watt P.W., Murdoch G., Rennie M.J. (1987). Decrease in human quadriceps muscle protein turnover consequent upon leg immobilization. Clin. Sci. (Lond.).

[bib26] Glover E.I., Phillips S.M., Oates B.R., Tang J.E., Tarnopolsky M.A., Selby A., Smith K., Rennie M.J. (2008). Immobilization induces anabolic resistance in human myofibrillar protein synthesis with low and high dose amino acid infusion. J. Physiol..

[bib27] Gnerer J.P., Kreber R.A., Ganetzky B. (2006). Wasted away, a Drosophila mutation in triosephosphate isomerase, causes paralysis, neurodegeneration, and early death. Proc. Natl. Acad. Sci. USA.

[bib28] Gobbi A., Jurman G. (2015). A null model for Pearson coexpression networks. PLOS ONE.

[bib29] Holloway T.M., Snijders T., Van Kranenburg J., Van Loon L.J.C., Verdijk L.B. (2018). Temporal Response of Angiogenesis and Hypertrophy to Resistance Training in Young Men. Med. Sci. Sports Exerc..

[bib30] Hwangbo C., Wu J., Papangeli I., Adachi T., Sharma B., Park S., Zhao L., Ju H., Go G.-w., Cui G. (2017). Endothelial APLNR regulates tissue fatty acid uptake and is essential for apelin’s glucose-lowering effects. Sci. Transl. Med..

[bib31] Keenan A.B., Jenkins S.L., Jagodnik K.M., Koplev S., He E., Torre D., Wang Z., Dohlman A.B., Silverstein M.C., Lachmann A. (2018). The Library of Integrated Network-Based Cellular Signatures NIH Program: System-Level Cataloging of Human Cells Response to Perturbations. Cell Syst..

[bib80] Keller P., Vollaard N.B.J., Gustafsson T., Gallagher I.J., Sundberg C.J., Rankinen T., Britton S.L., Bouchard C., Koch L.G., Timmons J.A. (2011). A transcriptional map of the impact of endurance exercise training on skeletal muscle phenotype. Journal of Applied Physiology.

[bib32] Kocabas A., Duarte T., Kumar S., Hynes M.A. (2015). Widespread Differential Expression of Coding Region and 3′ UTR Sequences in Neurons and Other Tissues. Neuron.

[bib33] Kosek D.J., Kim J.S., Petrella J.K., Cross J.M., Bamman M.M. (2006). Efficacy of 3 days/wk resistance training on myofiber hypertrophy and myogenic mechanisms in young vs. older adults. J. Appl. Physiol..

[bib34] Langmead B., Salzberg S.L. (2012). Fast gapped-read alignment with Bowtie 2. Nat. Methods.

[bib35] Li M., Verdijk L.B., Sakamoto K., Ely B., van Loon L.J.C.C., Musi N. (2012). Reduced AMPK-ACC and mTOR signaling in muscle from older men, and effect of resistance exercise. Mech. Ageing Dev..

[bib36] Li J.J., Biggin M.D. (2015). Gene expression. Statistics requantitates the central dogma. Science.

[bib37] Liu D., Sartor M.A., Nader G.A., Gutmann L., Treutelaar M.K., Pistilli E.E., Iglayreger H.B., Burant C.F., Hoffman E.P., Gordon P.M. (2010). Skeletal muscle gene expression in response to resistance exercise: sex specific regulation. BMC Genomics.

[bib38] MacInnis M.J., McGlory C., Gibala M.J., Phillips S.M. (2017). Investigating human skeletal muscle physiology with unilateral exercise models: when one limb is more powerful than two. Appl. Physiol. Nutr. Metab..

[bib39] Mayr C. (2017). Regulation by 3′-Untranslated Regions. Annu. Rev. Genet..

[bib84] McDonald M.L.N., Won S., Mattheisen M., Castaldi P.J., Cho M.H., Rutten M., Yip W., Rennard S.I., Lomas D.A., Wouters E.F.M. (2017). Body mass index change in gastrointestinal cancer and chronic obstructive pulmonary disease is associated with Dedicator of Cytokinesis 1. Journal of Cachexia Sarcopenia and Muscle.

[bib40] Melov S., Tarnopolsky M.A., Beckman K., Felkey K., Hubbard A. (2007). Resistance exercise reverses aging in human skeletal muscle. PLOS ONE.

[bib41] Miotto P.M., McGlory C., Bahniwal R., Kamal M., Phillips S.M., Holloway G.P. (2019). Supplementation with dietary ω-3 mitigates immobilization-induced reductions in skeletal muscle mitochondrial respiration in young women. FASEB J..

[bib42] Mitchell C.J., Churchward-Venne T.A., Parise G., Bellamy L., Baker S.K., Smith K., Atherton P.J., Phillips S.M. (2014). Acute post-exercise myofibrillar protein synthesis is not correlated with resistance training-induced muscle hypertrophy in young men. PLOS ONE.

[bib43] Moghei M., Tavajohi-Fini P., Beatty B., Adegoke O.A.J. (2016). Ketoisocaproic acid, a metabolite of leucine, suppresses insulin-stimulated glucose transport in skeletal muscle cells in a BCAT2-dependent manner. Am. J. Physiol. Cell Physiol..

[bib81] Morton R.W., Murphy K.T., McKellar S.R., Schoenfeld B.J., Henselmans M., Helms E., Aragon A.A., Devries M.C., Banfield L., Krieger J.W., Phillips S.M. (2018). A systematic review, meta-analysis and meta-regression of the effect of protein supplementation on resistance training-induced gains in muscle mass and strength in healthy adults. British Journal of Sports Medicine.

[bib44] Morton R.W., Oikawa S.Y., Wavell C.G., Mazara N., McGlory C., Quadrilatero J., Baechler B.L., Baker S.K., Phillips S.M. (2016). Neither load nor systemic hormones determine resistance training-mediated hypertrophy or strength gains in resistance-trained young men. J. Appl. Physiol..

[bib45] Peter I., Papandonatos G.D., Belalcazar L.M., Yang Y., Erar B., Jakicic J.M., Unick J.L., Balasubramanyam A., Lipkin E.W., Delahanty L.M., Look AHEAD Research Group (2014). Genetic modifiers of cardiorespiratory fitness response to lifestyle intervention. Med. Sci. Sports Exerc..

[bib46] Phillips S.M., McGlory C. (2014). CrossTalk proposal: the dominant mechanism causing disuse muscle atrophy is decreased protein synthesis. J. Physiol..

[bib47] Phillips B.E., Williams J.P., Gustafsson T., Bouchard C., Rankinen T., Knudsen S., Smith K., Timmons J.A., Atherton P.J. (2013). Molecular networks of human muscle adaptation to exercise and age. PLOS Genet..

[bib48] Phillips B., Kelly B., Lilja M., Ponce-González J., Brogan R., Morris D., Gustafsson T., Kraus W., Atherton P., Vollaard N. (2017). A practical and time-efficient high-intensity interval training programme modifies cardio-metabolic risk-factors in adults with risk-factors for type II diabetes. Front. Endocrinol..

[bib49] Pillon N.J., Gabriel B.M., Dollet L., Smith J.A.B., Sardón Puig L., Botella J., Bishop D.J., Krook A., Zierath J.R. (2020). Transcriptomic profiling of skeletal muscle adaptations to exercise and inactivity. Nat. Commun..

[bib50] Powers S.K., Wiggs M.P., Duarte J.A., Zergeroglu A.M., Demirel H.A. (2012). Mitochondrial signaling contributes to disuse muscle atrophy. Am. J. Physiol. Endocrinol. Metab..

[bib51] Raue U., Trappe T.A., Estrem S.T., Qian H.-R., Helvering L.M., Smith R.C., Trappe S. (2012). Transcriptome signature of resistance exercise adaptations: mixed muscle and fiber type specific profiles in young and old adults. J. Appl. Physiol (1985).

[bib52] Roberts M.D., Haun C.T., Mobley C.B., Mumford P.W., Romero M.A., Roberson P.A., Vann C.G., McCarthy J.J. (2018). Physiological differences between low versus high skeletal muscle hypertrophic responders to resistance exercise training: current perspectives and future research directions. Front. Physiol..

[bib53] Robinson M.M., Dasari S., Konopka A.R., Johnson M.L., Manjunatha S., Esponda R.R., Carter R.E., Lanza I.R., Nair K.S. (2017). Enhanced Protein Translation Underlies Improved Metabolic and Physical Adaptations to Different Exercise Training Modes in Young and Old Humans. Cell Metab..

[bib54] Sancak Y., Bar-Peled L., Zoncu R., Markhard A.L., Nada S., Sabatini D.M. (2010). Ragulator-Rag complex targets mTORC1 to the lysosomal surface and is necessary for its activation by amino acids. Cell.

[bib55] Sandri M., Barberi L., Bijlsma A.Y., Blaauw B., Dyar K.A., Milan G., Mammucari C., Meskers C.G.M., Pallafacchina G., Paoli A. (2013). Signalling pathways regulating muscle mass in ageing skeletal muscle: the role of the IGF1-Akt-mTOR-FoxO pathway. Biogerontology.

[bib85] Schneider C.A., Rasband W.S., Eliceiri K.W. (2012). NIH Image to ImageJ: 25 years of Image Analysis. Nature Methods.

[bib56] Schönbrodt F.D., Perugini M. (2013). At what sample size do correlations stabilize?. J. Res. Pers..

[bib57] Shalek A.K., Satija R., Adiconis X., Gertner R.S., Gaublomme J.T., Raychowdhury R., Schwartz S., Yosef N., Malboeuf C., Lu D. (2013). Single-cell transcriptomics reveals bimodality in expression and splicing in immune cells. Nature.

[bib58] Silventoinen K., Magnusson P.K.E., Tynelius P., Kaprio J., Rasmussen F. (2008). Heritability of body size and muscle strength in young adulthood: a study of one million Swedish men. Genet. Epidemiol..

[bib59] Song W.M., Zhang B. (2015). Multiscale Embedded Gene Co-expression Network Analysis. PLOS Comput. Biol..

[bib60] Sood S., Gallagher I.J., Lunnon K., Rullman E., Keohane A., Crossland H., Phillips B.E., Cederholm T., Jensen T., van Loon L.J.C. (2015). A novel multi-tissue RNA diagnostic of healthy ageing relates to cognitive health status. Genome Biol..

[bib61] Sood S., Szkop K.J., Nakhuda A., Gallagher I.J., Murie C., Brogan R.J., Kaprio J., Kainulainen H., Atherton P.J., Kujala U.M. (2016). iGEMS: an integrated model for identification of alternative exon usage events. Nucleic Acids Res..

[bib62] Stein C.S., Jadiya P., Zhang X., McLendon J.M., Abouassaly G.M., Witmer N.H., Anderson E.J., Elrod J.W., Boudreau R.L. (2018). Mitoregulin: A lncRNA-Encoded Microprotein that Supports Mitochondrial Supercomplexes and Respiratory Efficiency. Cell Rep..

[bib63] Subramanian A., Narayan R., Corsello S.M., Peck D.D., Natoli T.E., Lu X., Gould J., Davis J.F., Tubelli A.A., Asiedu J.K. (2017). A Next Generation Connectivity Map: L1000 Platform and the First 1,000,000 Profiles. Cell.

[bib64] Sweeney T.E., Haynes W.A., Vallania F., Ioannidis J.P., Khatri P. (2017). Methods to increase reproducibility in differential gene expression via meta-analysis. Nucleic Acids Res..

[bib65] Timmons J.A. (2011). Variability in training-induced skeletal muscle adaptation. J. Appl. Physiol. (1985).

[bib66] Timmons J.A., Larsson O., Jansson E., Fischer H., Gustafsson T., Greenhaff P.L., Ridden J., Rachman J., Peyrard-Janvid M., Wahlestedt C., Sundberg C.J. (2005). Human muscle gene expression responses to endurance training provide a novel perspective on Duchenne muscular dystrophy. FASEB J..

[bib67] Timmons J.A., Norrbom J., Schéele C., Thonberg H., Wahlestedt C., Tesch P. (2006). Expression profiling following local muscle inactivity in humans provides new perspective on diabetes-related genes. Genomics.

[bib68] Timmons J.A., Knudsen S., Rankinen T., Koch L.G., Sarzynski M., Jensen T., Keller P., Scheele C., Vollaard N.B.J., Nielsen S. (2010). Using molecular classification to predict gains in maximal aerobic capacity following endurance exercise training in humans. J. Appl. Physiol..

[bib69] Timmons J.A., Szkop K.J., Gallagher I.J. (2015). Multiple sources of bias confound functional enrichment analysis of global -omics data. Genome Biol..

[bib70] Timmons J.A., Atherton P.J., Larsson O., Sood S., Blokhin I.O., Brogan R.J., Volmar C.H., Josse A.R., Slentz C., Wahlestedt C. (2018). A coding and non-coding transcriptomic perspective on the genomics of human metabolic disease. Nucleic Acids Res..

[bib71] Timmons J.A., Volmar C.H., Crossland H., Phillips B.E., Sood S., Janczura K.J., Törmäkangas T., Kujala U.M., Kraus W.E., Atherton P.J., Wahlestedt C. (2019). Longevity-related molecular pathways are subject to midlife “switch” in humans. Aging Cell.

[bib72] Tusher V.G., Tibshirani R., Chu G. (2001). Significance analysis of microarrays applied to the ionizing radiation response. Proc. Natl. Acad. Sci. USA.

[bib73] van Dam S., Craig T., de Magalhães J.P. (2015). GeneFriends: a human RNA-seq-based gene and transcript co-expression database. Nucleic Acids Res..

[bib74] Vinel C., Lukjanenko L., Batut A., Deleruyelle S., Pradère J.-P., Le Gonidec S., Dortignac A., Geoffre N., Pereira O., Karaz S. (2018). The exerkine apelin reverses age-associated sarcopenia. Nat. Med..

[bib75] von Haehling S., Morley J.E., Anker S.D. (2012). From muscle wasting to sarcopenia and myopenia: update 2012. J. Cachexia Sarcopenia Muscle.

[bib76] Wang X., Kang D.D., Shen K., Song C., Lu S., Chang L.C., Liao S.G., Huo Z., Tang S., Ding Y. (2012). An R package suite for microarray meta-analysis in quality control, differentially expressed gene analysis and pathway enrichment detection. Bioinformatics.

[bib77] Wilkinson D.J., Franchi M.V., Brook M.S., Narici M.V., Williams J.P., Mitchell W.K., Szewczyk N.J., Greenhaff P.L., Atherton P.J., Smith K. (2014). A validation of the application of D(2)O stable isotope tracer techniques for monitoring day-to-day changes in muscle protein subfraction synthesis in humans. Am. J. Physiol. Endocrinol. Metab..

[bib78] Yasuda N., Glover E.I., Phillips S.M., Isfort R.J., Tarnopolsky M.A. (2005). Sex-based differences in skeletal muscle function and morphology with short-term limb immobilization. J. Appl. Physiol..

