# Supplementary material for: Molecular Transducers of Human Skeletal Muscle Remodeling under Different Loading States
Source: Cell Rep. 2020 Aug 4;32(5):107980. doi: 10.1016/j.celrep.2020.107980 (PMC7408494; doi:10.1016/j.celrep.2020.107980)
Supplement: Document S1. Figures S1–S7 and Tables S1 and S2 [file mmc1.pdf]

Cell Reports, Volume 32

Supplemental Information

Molecular Transducers of Human Skeletal Muscle Remodeling under Different Loading States

Tanner Stokes, James A. Timmons, Hannah Crossland, Thomas R. Tripp, Kevin Murphy, Chris McGlory, Cameron J. Mitchell, Sara Y. Oikawa, Robert W. Morton, Bethan E. Phillips, Steven K. Baker, Phillip J. Atherton, Claes Wahlestedt, and Stuart M. Phillips

Supplementary Figures and Tables

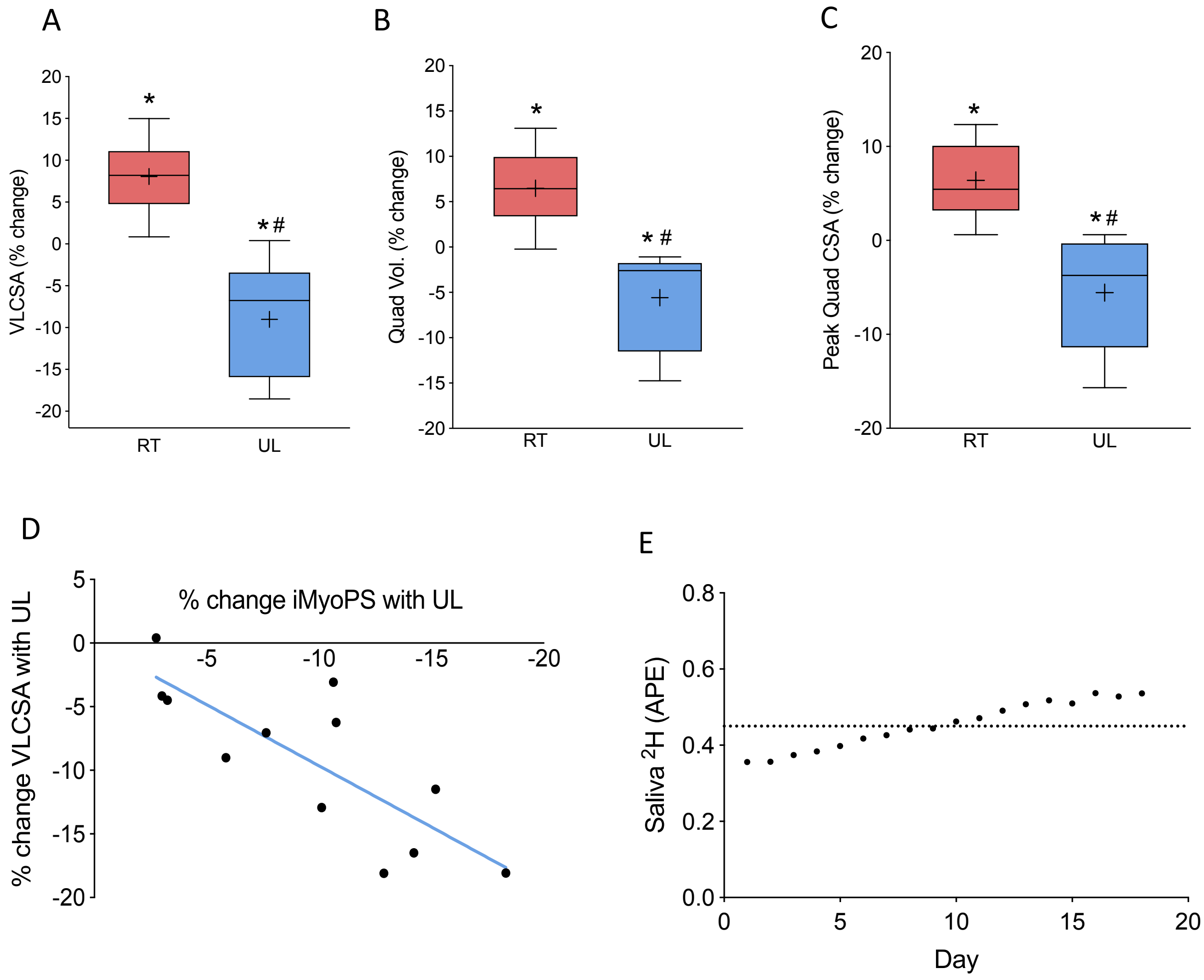

Figure S1. Muscle atrophy proceeds much more rapidly than muscle hypertrophy and is related to the change in muscle protein synthesis, related to Figure 2.

(A) Percentage change in mid-thigh *vastus lateralis* cross sectional area (VLCSA) following 10 weeks of resistance training (RT) and 2 weeks of unloading (UL); n=12.

(B) Percentage change in quadriceps volume following 10 weeks of RT and 2 weeks of UL, n=12.

(C) Percentage change in peak-quadriceps CSA following 10 weeks of RT and 2 weeks of UL, n=12.

(D) The linear relationship between changes in iMyoPS in response to 2 weeks of UL and the corresponding reduction of VLCSA in the same limb (Pearson's r=0.8, $p<0.05$, caveat with small sample size acknowledged despite the high probability of a causal relationship between these two variables); n=12.

(E) Average deuterium enrichment in saliva during the periods over which iMyoPS was assessed. The horizontal dotted line shows the global average from day 1 (i.e. after loading) until day 20; n=12.

For box and whisker plots, the boxes include the 25th, 50th and 75th quartiles and whiskers represent the maximum and minimum values. The mean value is depicted by the '+' symbol.

*denotes statistically different from Pre; # statistically different from RT value ($p<0.05$).

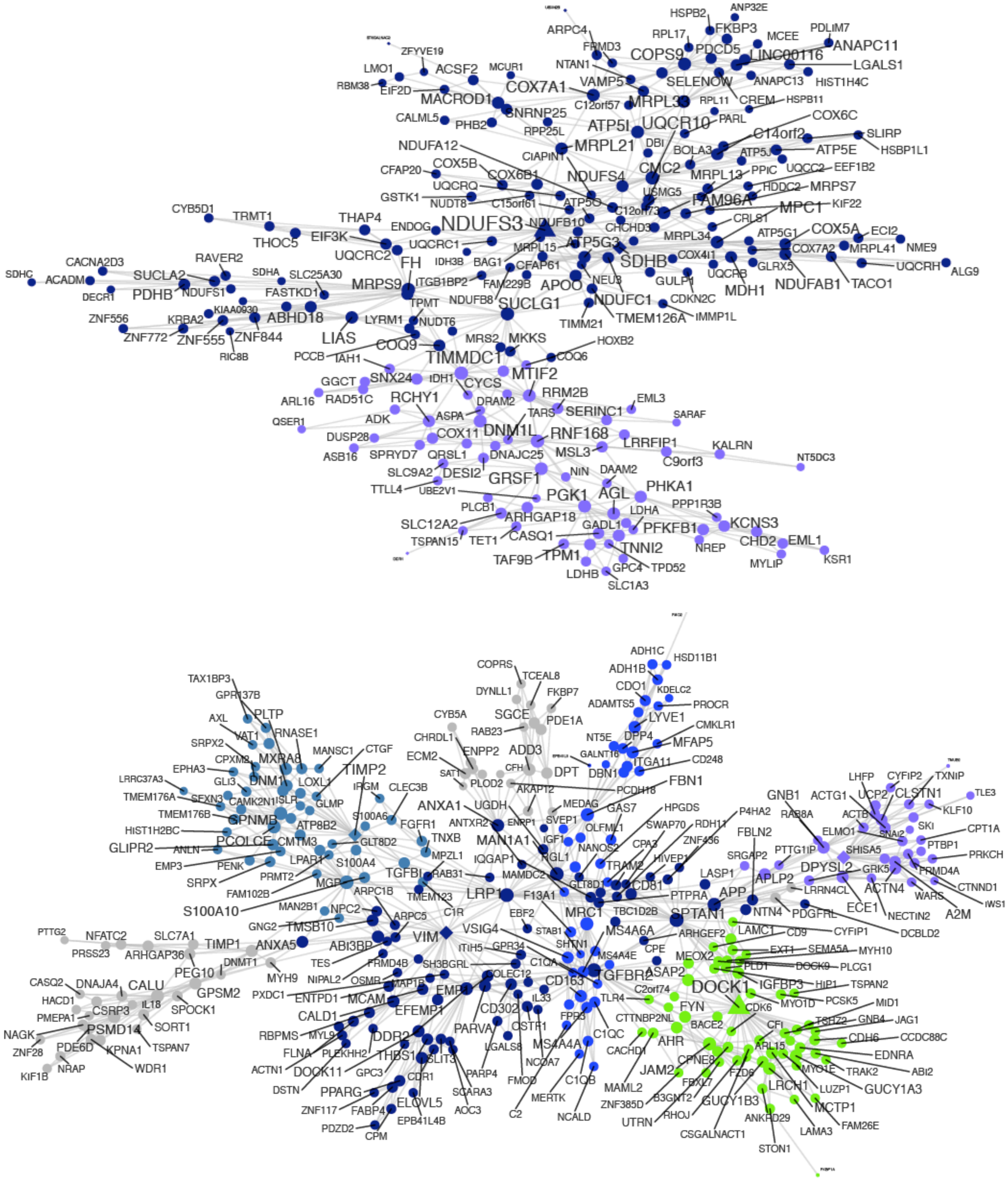

Figure S2. HypAt-regulated genes form functional networks in a large human muscle tissue biobank, related to Supplemental Data S1 and Figure 3.

Using the HypAT FL-ENST regulated transcripts (FDR <5%) as input into Megena (FDR <1% spearman correlation; p<0.01 for module significance, p<0.01 for network connectivity and 10,000 permutations for calculating FDR and connectivity p-values), top distinct planar filtered networks were identified that centered around NDUFS3 (top panel; enriched in genes relating to mitochondrial biology) and around DOCK1 (bottom

panel, enriched in genes related to extracellular matrix remodeling) using a large independent skeletal muscle data set (n=187).

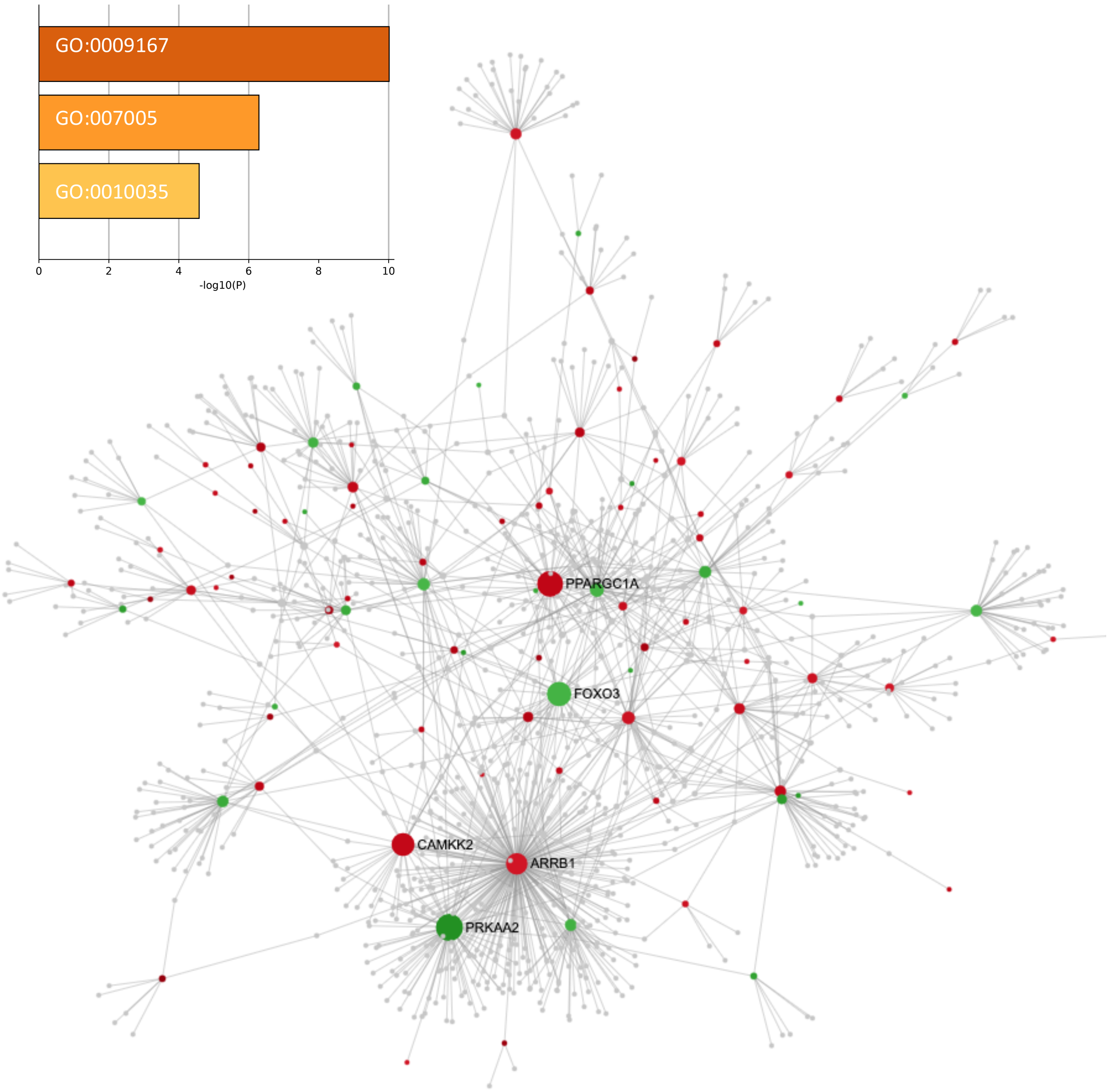

Figure S3. Proteome constrained network modeling reveals growth regulating pathways, related to Figure 3 and Table S2.
HypAT genes that correlated with lean mass gains in independent cohorts were used as input to characterize tissue-specific protein-protein interactions (PPI) using www.networkanalyst.ca and a 10th percentile threshold. A first-order network of protein-protein interactions is presented. FOXO3 was negatively correlated with leg lean mass changes and the PPI contained 45 FOXO signaling pathway members (Kegg database), $1x10^{-13}$ FDR. Green circles represent negatively- and red, positively-correlated genes. Grey genes are members of the protein-protein interactome acting on the 141 HypAT genes regulated in proportion to gains in lean mass. **Inset**: bar graph generated using Metascape showing top enrichment clusters derived from the list of growth correlated genes. GO Terms: GO:0009167, Purine ribonucleoside monophosphate metabolic process; GO:0007005, Mitochondrion

organization; GO:0010035, Response to inorganic substance. Color of bars indicates the level of significance of the corresponding cluster. Genes related to muscle growth in independent data sets were found to be dominated by proteins related to mitochondrial biology and ribonucleoside monophosphate synthesis.

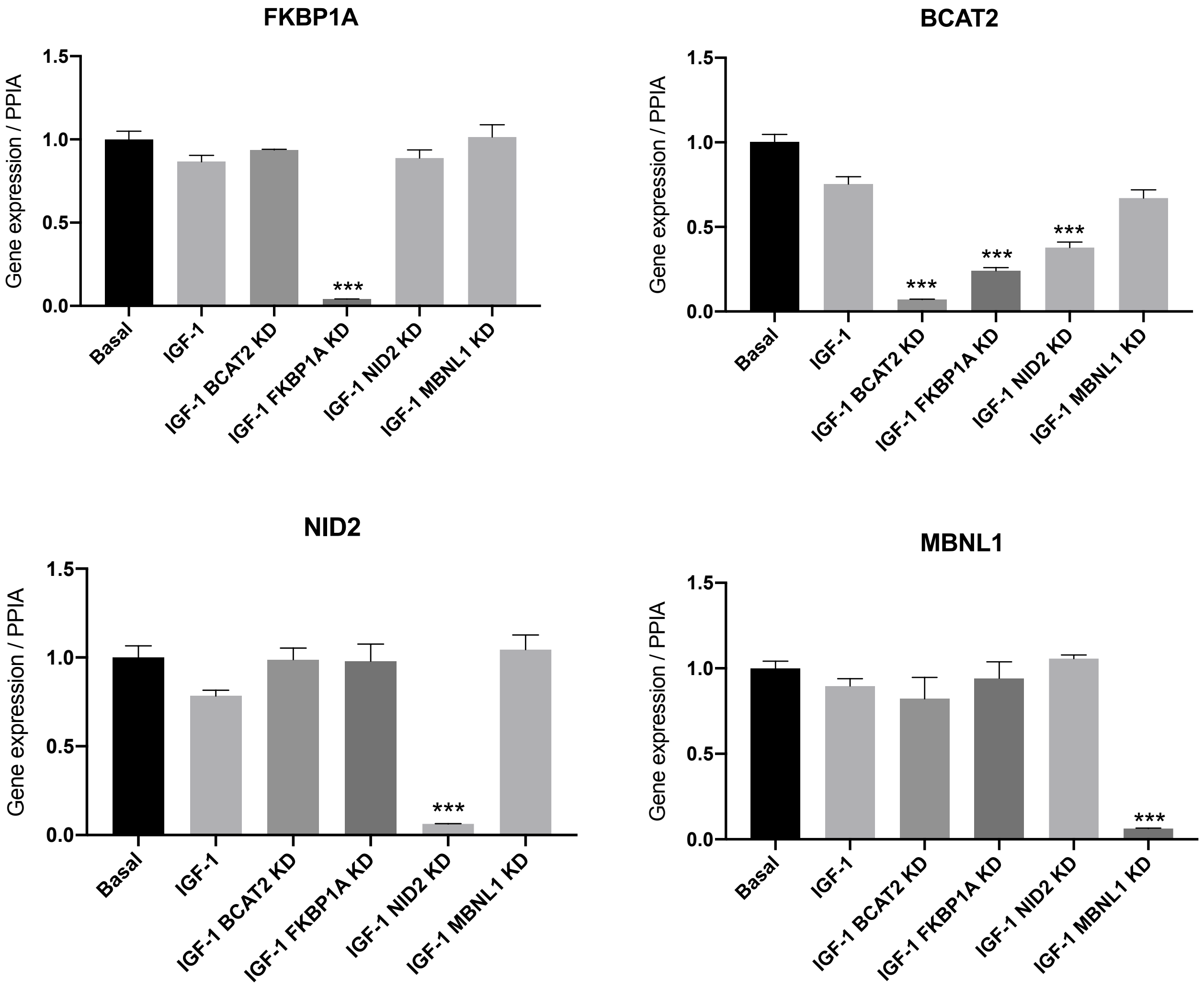

Figure S4. RNAi effectively reduced RNA expression of selected growth-correlated target genes by >90% in differentiated myotubes, related to Figure 4.
Normalized gene expression data from myotubes after knockdown with siRNA species against: FKBP1, BCAT2, MBNL1 and NID2. Cells were either untreated (set to 100%), treated with IGF-1 only, or treated with IGF-1 and with a pool of siRNA targeted against the gene listed. n=4; one-way ANOVA (*$P<0.05$, **$P<0.01$, ***$P<0.001$ versus IGF-1)

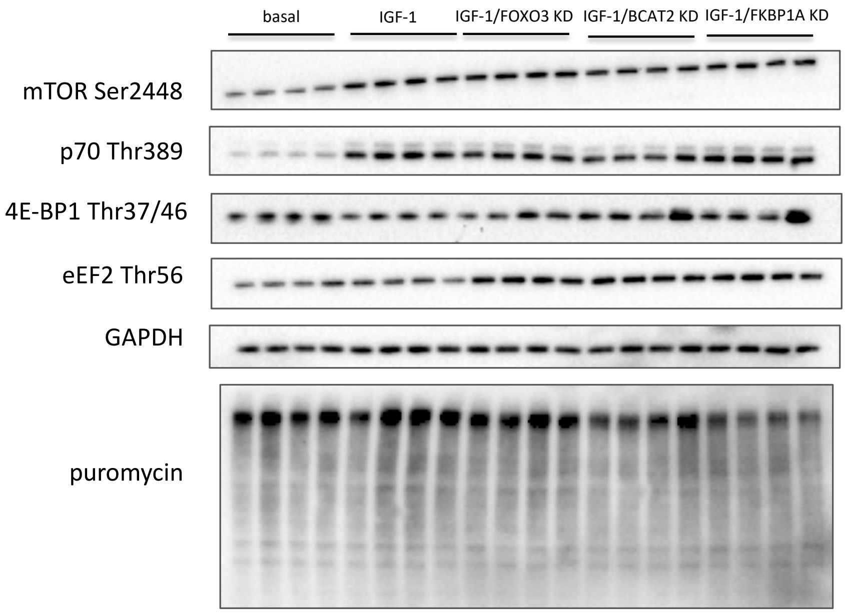

Lane order (n=4): basal, IGF-1, IGF-1/FOXO3 KD, IGF-1/BCAT2 KD, IGF-1/FKBP1A KD

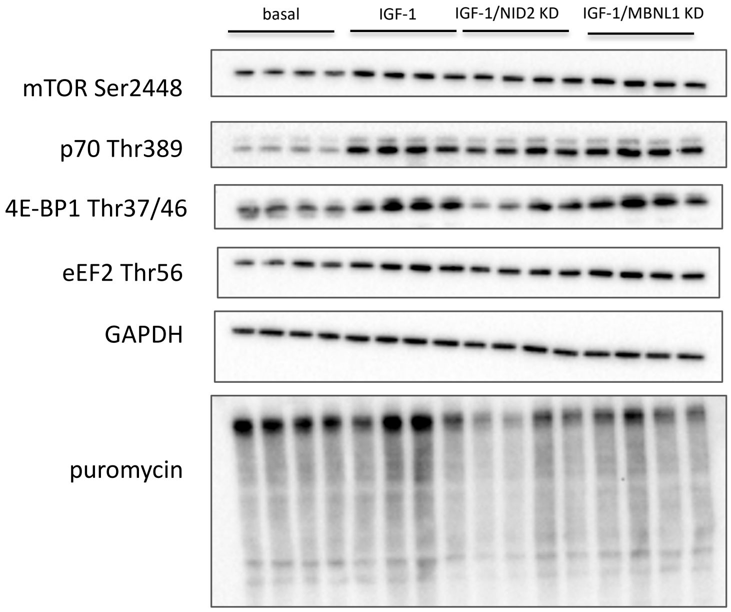

Lane order (n=4): basal, IGF-1, IGF-1/NID2 KD, IGF-1/MNBNL1 KD

Figure S5. Representative western blots of protein signaling data, related to Figure 4.
Lane order (n=4): basal (untreated), IGF-1, IGF-1/FOXO KD, IGF-1/BCAT2 KD, IGF-1/FKBP1a KD. Note: the RNAi tool against FOXO3 was inactive and so we did not, as originally planned, study FOXO3 as a control. However, western analyses were carried out prior to the discovery that FOXO3 siRNA was defective and thus included in the original gels.

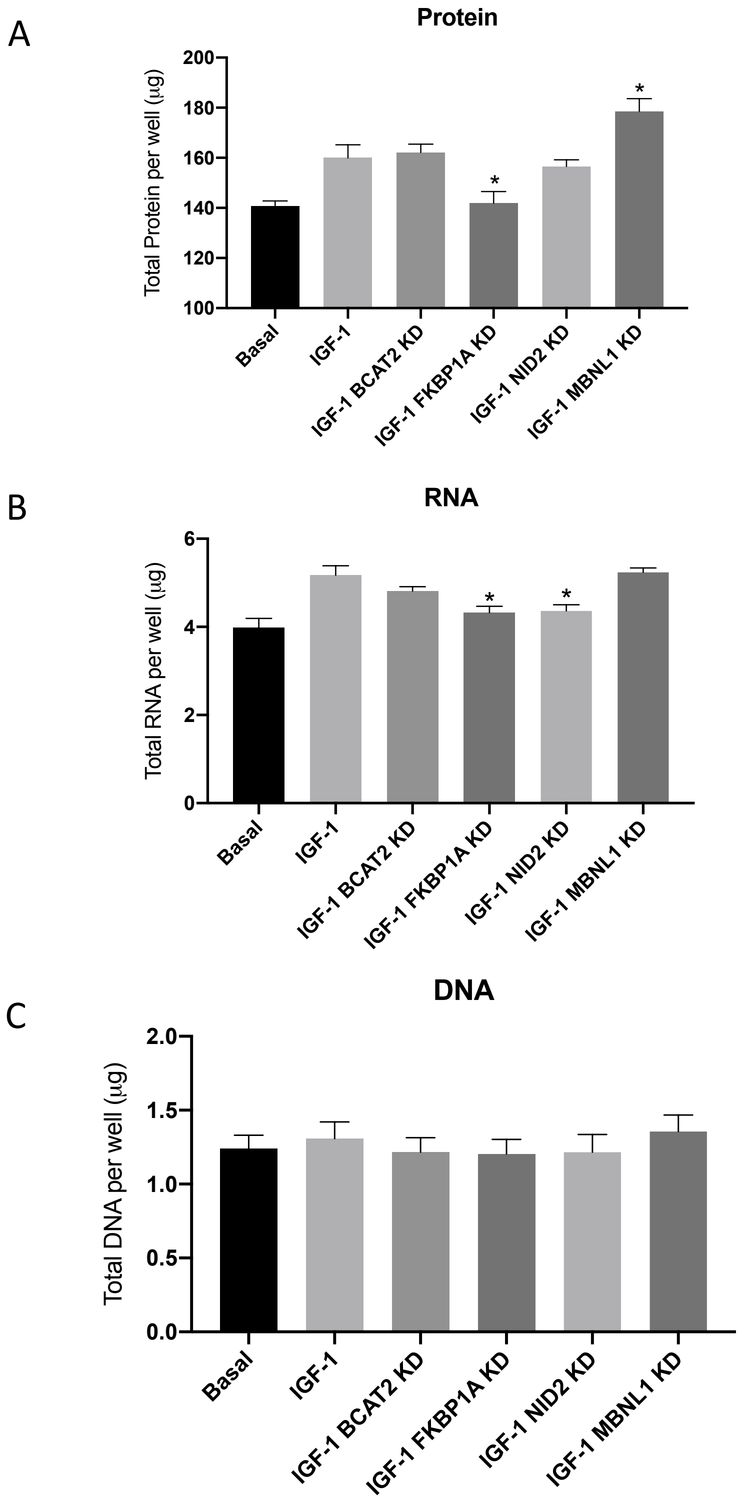

Figure S6. Total protein, RNA and DNA content after myotube treatment with or without IGF-1 and siRNA against selected gene targets, related to Figure 4.
Total protein (A), RNA (B) and DNA (C) after myotube treatment with IGF-1 in isolation or combined with siRNA against BCAT2, FKBP1A, NID2 or MBNL1. n=5; one-way ANOVA (*P<0.05, versus IGF-1).

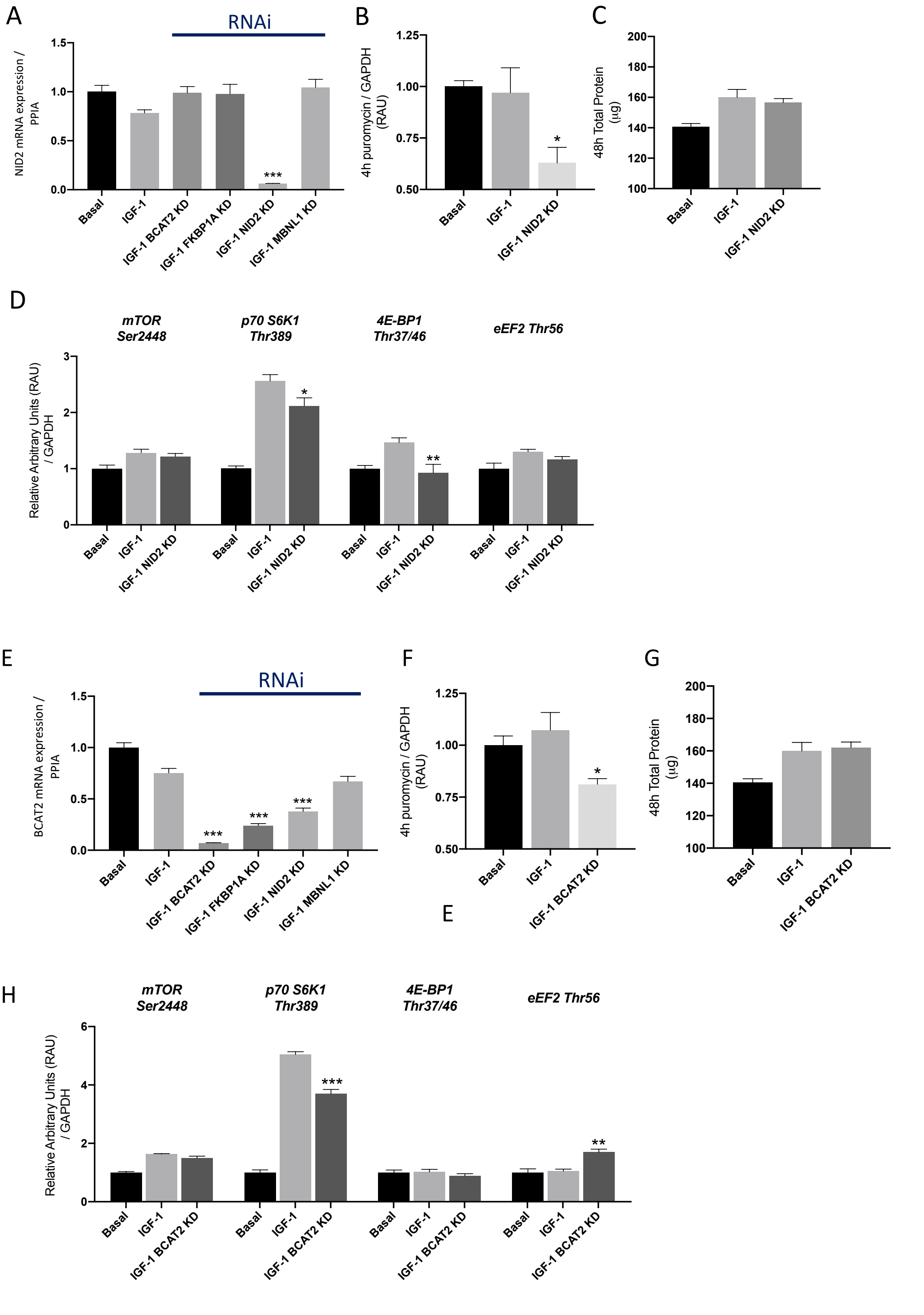

Figure S7. Knockdown of growth-correlated genes in myotubes alters protein synthesis and regulates signaling cascades that influence protein translation, related to Figure 4.

(A – D) The effects of targeting NID2 with siRNA on NID2 mRNA expression (A), 4hr puromycin signal (B), 48hr total protein (C) and protein content of various proteins thought to be involved in muscle growth.
(E – H) The effects of targeting BCAT2 with siRNA on BCAT2 mRNA expression (A), 4hr puromycin signal (B), 48hr total protein (C) and protein content of various proteins thought to be involved in muscle growth.

Supplementary Tables

Table S1. HypAt Participant Characteristics, related to Figure 1, 2, S1 and STAR Methods.

Parameter	Baseline	Week 5	Week 10
Age, y	20±3	-	-
Height, m	1.7±0.1	-	-
Mass, kg	71.2±12.2	71.2±11.4	70.8±11.2
BMI, $kg \cdot m^{-2}$	23.8±3.1	23.9±2.9	23.7±2.8
Leg Ext 1-RM, kg	53±12	-	77±12*
Leg Press 1-RM, kg	120±32	-	190±35*
Daily Steps	9900±5100	9100±2800	8000±3600
Activity, $kcal \cdot d^{-1}$	1012±477	970±403	965±437
Dietary Protein, $g \cdot kg \cdot d^{-1}$	1.5±0.9	1.4±0.7	1.6±0.9
En% Protein	17±6	19±5	18±4
En% CHO	49±15	52±7	54±10
En% Fat	32±12	31±7	28±10

Abbreviations: BMI, body mass index; 1-RM, one-repetition maximum; En%, energy percentage. CHO, carbohydrates. * significantly different from baseline, $p<0.05$. All data are presented as mean±SD.

Table S2. Steps for generating a proteome-constrained network plot, related to Table 1, STAR Methods, Figure S3 and Supplemental Data S2.

Steps	Link/File
Get list of genes for uploading to web-site	URL to Supplemental Data S2
Browse to network tool home page	www.networkanalyst.ca
Select 'Gene List Input' option	https://www.networkanalyst.ca/NetworkAnalyst/uploads/ListUploadView.xhtml
Select 'Human' and Official Gene Symbol	Pull-down lists
Open file containing gene list	Browse to location of 'Supplementary Data S2' file on your computer and open in text editor or Excel - then copy both columns
Upload gene list	Paste gene list and press upload and then press proceed
Choose Tissue-Specific PPI	Select 'skeletal muscle' from the pull-down menu and set the filter to 10
Define Network	Press proceed https://www.networkanalyst.ca/NetworkAnalyst/Secure/network/NetworkBuilder.xhtml)
Define network structure	Press 3D option within network plotting window. Then set 'view' to 'expression' and 'shading' = 'none'.
View 3D network	Place mouse cursor on any black space, hold-down mouse button and drag to rotate. Type any gene name into the 'search' function to highlight that gene. Zoom in and out to view network connections.
Explore pathway biology	Select database to query from top right-hand side of web page. E.g. Kegg. Choose all nodes and press submit

A step by step guide to generating a 3-dimentional plot of the 141 HypAT genes that correlated with lean mass gains across independent cohorts within the context of a muscle tissue-specific protein-protein interactions (PPI) network. Note, in November 2019, the analysis indicates FOXO3 was negatively correlated with leg lean mass changes and the PPI contains 45 FOXO signaling pathway members (Kegg database, $1x10^{-13}$ FDR). Green represents negatively and red positively correlated genes with in vivo changes in lean mass. Grey circles are members of the protein-protein interactome, acting on the 141 HypAt genes regulated in proportion to gains in lean mass, but themselves were not regulated at the RNA level in the present analysis. Any regulated gene, from the 141 identified, that was not part of the protein-protein interactome would not appear in this analysis as evidence for protein level interaction was a prerequisite for inclusion.
